# Supplementary material for: Nonlinear eco-evolutionary games with global environmental fluctuations and local environmental feedbacks
Source: PLoS Comput Biol. 2023 Jun 28;19(6):e1011269. doi: 10.1371/journal.pcbi.1011269 (PMC10335700; doi:10.1371/journal.pcbi.1011269)
Supplement: S1 Appendix — (PDF) [file pcbi.1011269.s001.pdf]

S1 Appendix. Stability of fixed points in static global environments

For any given w ($w \neq 1$), the dynamic equations of the eco-evolutionary system can be written as

$$\begin{cases} \dot{x} = x\,(1-x)\,\left(\frac{r_c\left(w(wx-x+1)^{N-1}-1\right)}{N(w-1)} - 1 - \frac{r_d\left((wx-x+1)^{N-1}-1\right)}{N(w-1)}\right) \\ \dot{r}_c = \epsilon\,(r_c-\alpha)\,(\beta-r_c)\,\left(-x\left(\frac{r_c\left(w(wx-x+1)^{N-1}-1\right)}{N(w-1)} - 1\right)\right. \\ \left. + \frac{r_d\theta(1-x)\left((wx-x+1)^{N-1}-1\right)}{N(w-1)}\right) \end{cases}. \quad (1)$$

Jacobian matrix of this system is

$$J = \begin{bmatrix} A & B \\ C & D \end{bmatrix}, \quad (2)$$

where

$$\begin{aligned}
A &= (2x-1)\,\left(\frac{r_d\left((wx-x+1)^{N-1}-1\right)}{N\,(w-1)} - \frac{r_c\left(w(wx-x+1)^{N-1}-1\right)}{N\,(w-1)} + 1\right) \\
&+ \frac{x\,(1-x)\,(r_c w - r_d)\,(N-1)\,(wx-x+1)^{N-2}}{N} \\
B &= \frac{x\,(1-x)\left(w(wx-x+1)^{N-1}-1\right)}{N\,(w-1)} \\
C &= \epsilon\,(r_c-\alpha)\,(\beta-r_c)\,\left(\frac{r_c\left(1-w(wx-x+1)^{N-1}\right)}{N\,(w-1)}\right. \\
&+ \frac{r_d\theta\left(1-(wx-x+1)^{N-1}\right)}{N\,(w-1)} + \frac{r_d\theta\,(N-1)\,(1-x)\,(wx-x+1)^{N-2}}{N} \\
&\left. - \frac{r_c wx\,(N-1)\,(wx-x+1)^{N-2}}{N} + 1\right) \\
D &= \epsilon\,(2r_c-\alpha-\beta)\,\left(x\left(\frac{r_c\left(w(wx-x+1)^{N-1}-1\right)}{N\,(w-1)} - 1\right)\right. \\
&\left. + \frac{r_d\theta\,(x-1)\,\left((wx-x+1)^{N-1}-1\right)}{N\,(w-1)}\right) \\
&+ \frac{\epsilon x\left(1-w(wx-x+1)^{N-1}\right)\,(r_c-\alpha)\,(\beta-r_c)}{N\,(w-1)}
\end{aligned} \quad (3)$$

Solving $\dot{x}=0$ and $\dot{r}_c=0$, we can derive the following fixed points.

(1) $x^*=0$

$$J\left(x^*=0,r_c\right)=\begin{bmatrix} \frac{r_c}{N}-1 & 0 \\ \epsilon\left(r_c-\frac{3}{2}\right)\left(\frac{7}{2}-r_c\right)\left(\frac{r_d\theta(N-1)}{N}-\frac{r_c}{N}+1\right) & 0 \end{bmatrix} \tag{4}$$

Eigenvalues are $\lambda_1=\frac{r_c-(N+1)}{N}<0$ (since $r_c\leq\alpha<N$), $\lambda_2=0$. So this fixed point is stable.

(2) $x^*=1, r_c^*=\alpha$

$$J\left(1,\alpha\right)=\begin{pmatrix} \frac{r_d\left(w^{N-1}-1\right)-\alpha\left(w^N-1\right)}{N(w-1)}+1 & 0 \\ 0 & \epsilon\left(\beta-\alpha\right)\left(1-\frac{\alpha\left(w^N-1\right)}{N(w-1)}\right) \end{pmatrix} \tag{5}$$

Eigenvalues are $\lambda_1=\frac{r_d\left(w^{N-1}-1\right)-\alpha\left(w^N-1\right)}{N(w-1)}+1$ and $\lambda_2=\epsilon\left(\beta-\alpha\right)\left(1-\frac{\alpha\left(w^N-1\right)}{N(w-1)}\right)$. When $\frac{\alpha\left(w^N-1\right)}{N(w-1)}>1$ and $r_d<\frac{\alpha\sum\limits_{k=0}^{N-1}w^k-N}{\sum\limits_{k=0}^{N-2}w^k}=\frac{\alpha\left(w^N-1\right)-N(w-1)}{\left(w^{N-1}-1\right)}=r_d^*$, we have $\lambda_1<0$ and $\lambda_2<0$ and this fixed point is stable.

(3) $x^*=1, r_c^*=\beta$

$$J\left(1,\beta\right)=\begin{pmatrix} \frac{r_d\left(w^{N-1}-1\right)}{N(w-1)}-\frac{\beta\left(w^N-1\right)}{N(w-1)}+1 & 0 \\ 0 & \epsilon\left(\beta-\alpha\right)\left(\frac{\beta\left(w^N-1\right)}{N(w-1)}-1\right) \end{pmatrix} \tag{6}$$

Eigenvalues are $\lambda_1=\frac{r_d\left(w^{N-1}-1\right)}{N(w-1)}-\frac{\beta\left(w^N-1\right)}{N(w-1)}+1$ and $\lambda_2=\epsilon\left(\beta-\alpha\right)\left(\frac{\beta\left(w^N-1\right)}{N(w-1)}-1\right)$. We can conclude that it is impossible for λ_1 and λ_2 to be negative simultaneously, the proof of which is as follows: If $\lambda_2<0$, we have $\frac{\beta\left(w^N-1\right)}{N(w-1)}<1$. Furthermore, if $\lambda_1<0$, we have $\frac{r_d\left(w^{N-1}-1\right)}{N(w-1)}<\frac{\beta\left(w^N-1\right)}{N(w-1)}-1<0$. Then we have $r_d<0$, which is contradicted with the real situation $r_d\geq 0$. Thus, there is at least one eigenvalue no less than 0, which indicates that the fixed point is unstable.

(4) $x^*=1, r_c^*=\frac{N(w-1)}{w^N-1}$ (If $\alpha<r_c^*<\beta$, the fixed point is in domain of definition)

$$J\left(x^*,r_c^*\right)=\begin{bmatrix} A & 0 \\ C & D \end{bmatrix} \tag{7}$$

where

$$\begin{aligned} A &= \frac{r_d\left(w^{N-1}-1\right)}{N\left(w-1\right)}-\frac{r_c^*\left(w^N-1\right)}{N\left(w-1\right)}+1=\frac{r_d\left(w^{N-1}-1\right)}{N\left(w-1\right)}>0 \\ C &= \epsilon\left(\alpha-r_c\right)\left(\beta-r_c\right)\left(\frac{r_c^*\left(w^N-1\right)}{N\left(w-1\right)}+\frac{r_d\theta\left(w^{N-1}-1\right)}{N\left(w-1\right)}+\frac{r_c^*w^{N-1}\left(N-1\right)}{N}-1\right) \\ D &= \frac{\epsilon\left(\alpha-r_c^*\right)\left(\beta-r_c^*\right)\left(w^N-1\right)}{N\left(w-1\right)}<0 \end{aligned} \tag{8}$$

Eigenvalues are $\lambda_1 = \frac{r_d\left(w^{N-1}-1\right)}{N(w-1)} > 0$ and $\lambda_2 = \frac{\epsilon(\alpha-r_c^*)(\beta-r_c^*)\left(w^N-1\right)}{N(w-1)} < 0$. This fixed point is a saddle point.

(5) $x^* = \frac{\left(\frac{\alpha-r_d+N(w-1)}{\alpha w-r_d}\right)^{\frac{1}{N-1}}-1}{w-1}, r_c^* = \alpha$ (If $0 < x^* < 1$, the fixed point is in domain of definition)

$$J\left(x^*, \alpha\right) = \left[\begin{array}{cc} A & B \\ 0 & D \end{array}\right] \tag{9}$$

where

$$\begin{aligned} A &= \frac{x^*\left(1-x^*\right)\left(\alpha w - r_d\right)\left(N-1\right)\left(wx^*-x^*+1\right)^{N-2}}{N} \\ B &= \frac{x^*\left(1-x^*\right)\left(w\left(wx^*-x^*+1\right)^{N-1}-1\right)}{N\left(w-1\right)} \\ D &= \epsilon\left(\beta-\alpha\right)\left(-x^*\left(\frac{\alpha\left(w\left(wx^*-x^*+1\right)^{N-1}-1\right)}{N\left(w-1\right)}-1\right)\right. \\ &\left.+\frac{r_d\theta\left(1-x^*\right)\left(\left(wx^*-x^*+1\right)^{N-1}-1\right)}{N\left(w-1\right)}\right) \end{aligned} \tag{10}$$

Eigenvalues are $\lambda_1 = A$ and $\lambda_2 = D$. Here we consider the two following situations: (1) $w > 1$ and (2) $w < 1$.

Case $w > 1$: Due to $r_d < \alpha < w\alpha$, we have $\alpha w - r_d > 0$, which indicates $\lambda_1 > 0$. Therefore, this fixed point is unstable.

Case $w < 1$: Assume $\lambda_1 < 0$. This assumption leads to $\alpha w - r_d < 0$. Here we set $N = 4$. Considering $0 < x^* = \frac{\left(\frac{\alpha-r_d+N(w-1)}{\alpha w-r_d}\right)^{\frac{1}{N-1}}-1}{w-1} < 1$, we can derive $w^3 < \frac{\alpha-r_d+4(w-1)}{\alpha w-r_d} < 1$. By calculating the right half of the inequality, we have $\alpha - r_d + 4\left(w-1\right) > \alpha w - r_d$, i.e. $w > 1$. Contradictory appears, so the assumption does not hold, which means that this fixed point is unstable.

(6) $x^* = \frac{\left(\frac{\beta-r_d+N(w-1)}{\beta w-r_d}\right)^{\frac{1}{N-1}}-1}{w-1}, r_c^* = \beta$ (If $0 < x^* < 1$, the fixed point is in domain of definition)

Jacobian is similar to (5), one of whose eigenvalues is $\lambda_1 = A = \frac{x^*(1-x^*)(\beta w-r_d)(N-1)(wx^*-x^*+1)^{N-2}}{N} > 0$. Thus, this fixed point is unstable.

(7) Interior fixed point $\left(x^* = \frac{\theta}{\theta+1}, r_c^* = \frac{N(w-1)+r_d\left((wx^*-x^*+1)^{N-1}-1\right)}{w(wx^*-x^*+1)^{N-1}-1}\right)$ (If $\alpha \le r_c^* \le \beta$, the fixed point is in domain of definition)

$$J\left(x^*, r_c^*\right) = \left[\begin{array}{cc} A & B \\ C & D \end{array}\right] \tag{11}$$

where

$$A = \frac{x^* (1 - x^*)\ (r_c^* w - r_d)\ (N - 1)\ (wx^* - x^* + 1)^{N-2}}{N}$$

$$B = \frac{x^* (1 - x^*) \left(w(wx^* - x^* + 1)^{N-1} - 1\right)}{N (w - 1)}$$

$$C = -f_c (x^*, r_c^*) - \theta f_d (x^*, r_c^*) - x f_c^{'} (x^*, r_c^*) + \theta (1 - x) f_d^{'} (x^*, r_c^*)$$

$$= - (1 + \theta) f_d (x^*, r_c^*) - x f_c^{'} (x^*, r_c^*) + \theta (1 - x) f_d^{'} (x^*, r_c^*)$$

$$= \epsilon (r_c^* - \alpha) (\beta - r_c^*) \left(\frac{(1 + \theta) r_d \left(1 - (wx^* - x^* + 1)^{N-1}\right)}{N (w - 1)} + [r_d \theta (1 - x^*) - r_c^* w x^*] \frac{(N - 1) (wx^* - x^* + 1)^{N-2}}{N} \right)$$

$$D = \epsilon (r_c^* - \alpha)\ (\beta - r_c^*) \frac{x^* \left(1 - w(wx^* - x^* + 1)^{N-1}\right)}{N (w - 1)} \tag{12}$$

This fixed point is stable if and only if the following two conditions holds:

$$\begin{cases} AD - BC > 0 \\ A + D < 0 \end{cases} \tag{13}$$

The first equality $AD - BC = \frac{\epsilon(r_c^* - \alpha)(\beta - r_c^*) x^* (1 - x^*)}{N^2} \frac{w(wx^* - x^* + 1)^{N-1} - 1}{w - 1} \frac{r_d (1 + \theta)\left((wx^* - x^* + 1)^{N-1} - 1\right)}{w - 1} > 0$ always hold. Thus this point is stable if and only if $A + D > 0$, which is equivalent to the following condition:

$$\epsilon > \epsilon^* \tag{14}$$

where

$$\begin{aligned} \epsilon^* &= \frac{(1 - x^*) (r_c^* w - r_d) (N - 1) (wx^* - x^* + 1)^{N-2} (w - 1)}{(r_c^* - \alpha) (\beta - r_c^*) \left[w(wx^* - x^* + 1)^{N-1} - 1\right]} \\ &= \frac{(r_c^* w - r_d) (N - 1) (w\theta + 1)^{N-2} (w - 1)}{(r_c^* - \alpha) (\beta - r_c^*) \left[w(w\theta + 1)^{N-1} - (1 + \theta)^{N-1}\right]} \end{aligned} \tag{15}$$

In conclusion, on the premise of $w \neq 1$, the stable fixed points and the conditions of stability are as follows:

(1) The fixed points on the vertical-axes ($x^* = 0$) are always stable.

(2) The fixed point ($x^* = 1, r_c^* = \alpha$) is stable when $\frac{\alpha\left(w^N - 1\right)}{N(w-1)} > 1$ and $r_d < \frac{\alpha\left(w^N - 1\right) - N(w-1)}{(w^{N-1} - 1)}$.

(3) The interior fixed point $\left(x^* = \frac{\theta}{\theta + 1}, r_c^* = \frac{N(w-1) + r_d\left((wx^* - x^* + 1)^{N-1} - 1\right)}{w(wx^* - x^* + 1)^{N-1} - 1}\right)$ is stable when $\epsilon > \frac{(1 - x^*)(r_c^* w - r_d)(N-1)(wx^* - x^* + 1)^{N-2}(w-1)}{(r_c^* - \alpha)(\beta - r_c^*)\left[w(wx^* - x^* + 1)^{N-1} - 1\right]}$.
