# Supplementary material for: Nonlinear eco-evolutionary games with global environmental fluctuations and local environmental feedbacks
Source: PLoS Comput Biol. 2023 Jun 28;19(6):e1011269. doi: 10.1371/journal.pcbi.1011269 (PMC10335700; doi:10.1371/journal.pcbi.1011269)
Supplement: S2 Appendix — We first consider a continuous function to describe the continuously changing global environment, which exhibits similar evolutionary trends to Fig 4. Then we consider a four-segment function to validate our explanations about the emergence of interior closed orbit in continuously changing global environments. (PDF) [file pcbi.1011269.s002.pdf]

S2 Appendix. Discretely varying and continuously changing global environments

In this part, we first consider continuous fluctuations modeled by a continuous function $w_3(t)$, which is simply given by

$$w_3(t) = 1 - 0.5\sin(at + \delta). \tag{1}$$

where a decides the time scales of global environmental fluctuations and δ modulates the initial phase. Note that $w_3(t)$ is bounded in $[0.5, 1.5]$ in order to balance the degree of discounting and synergy. In addition, for the convenience of comparison, we let the periods of $w_1(t)$ and $w_3(t)$ be equal, i.e., $T = \frac{\pi}{a}$. We also let the expectations of synergistically enhanced or discounted influence exerted by $w_1(t)$ and $w_3(t)$ in time interval T be equal. As a result, we set $w_1(t)$ change between $\frac{1}{T}\int_0^T(1-0.5\sin at)\,\mathrm{d}t \approx 0.7$ and $\frac{1}{T}\int_T^{2T}(1-0.5\sin at)\,\mathrm{d}t \approx 1.3$. Evolutionary trends similar to Fig 4 can be observed in a continuously changing global environment $w = w_3(t)$ (Fig S2.1). For easy to compare, we also set $\theta = 0.5$, $r_d = 0.6$ and $\delta = -\frac{\pi}{2}$. The thresholds corresponding to the maximum and minimum values of $w_3(t)$ are $\epsilon_{\max} = 5.29$ and $\epsilon_{\min} = 1.45$. Thus we choose $\epsilon = 1, 2.5, 6$ such that $1 < \epsilon_{\min} < 2.5 < \epsilon_{\max} < 6$. Likewise, the population will be occupied either by defectors or cooperators when ϵ is small. When ϵ is at an intermediate value, the cyclic evolution along an interior closed orbit arises from a rapidly changing global environment. When ϵ is large, such interior closed orbit occurs at all global time-scales.

We concentrate on the newly discovered phenomenon that the local game-environment dynamics can evolve cyclically under different periodic global environments (Fig S2.2). Due to the complexity of the non-autonomous system and the lack of theoretical insight, we display in detail the phase plane dynamics of local game-environment evolution, the typical dynamic trajectories with different initial conditions, and the corresponding time evolution of the frequency of cooperators x and the multiplication factor of cooperators r_c, in order to provide a clear view of the cyclic evolution. We set $\theta = 1$, $r_d = 1.2$, $a = 0.1$, $\delta = 0$ and choose $\epsilon = 4$ which is larger than the maximum threshold of ϵ^* with regard to $w_1(t)$ and $w_3(t)$. As interpreted in the main text, we find that the reason for the formation of cyclic evolution under discrete global environment $w_1(t)$ is that the two fixed points are in the mutual attraction domains (Fig S2.2b). In a continuously changing global environment described by $w_3(t)$, however, the fixed point also varies over time which is unreachable for the evolutionary process. Surprisingly, the interior closed yet irregular orbit still emerges.

We interpret our results by applying the limit thought: the evolution is always moving towards the nearest stable fixed point in each small time interval, and the dynamical trajectories are approximately the combinations of all such short paths connecting head to tail, the limit of which finally converge to a closed yet irregular orbit as we observed. We further consider a four-segment function $w_4(t)$ to validate our intuition. Specifically,

$$w_4(t) = \begin{cases} 1.3 & [t/T] = 4n \\ 1 & [t/T] = 4n+1 \\ 0.7 & [t/T] = 4n+2 \\ 1 & [t/T] = 4n+3 \end{cases}, n = 0, 1, 2, \cdots\cdots. \tag{2}$$

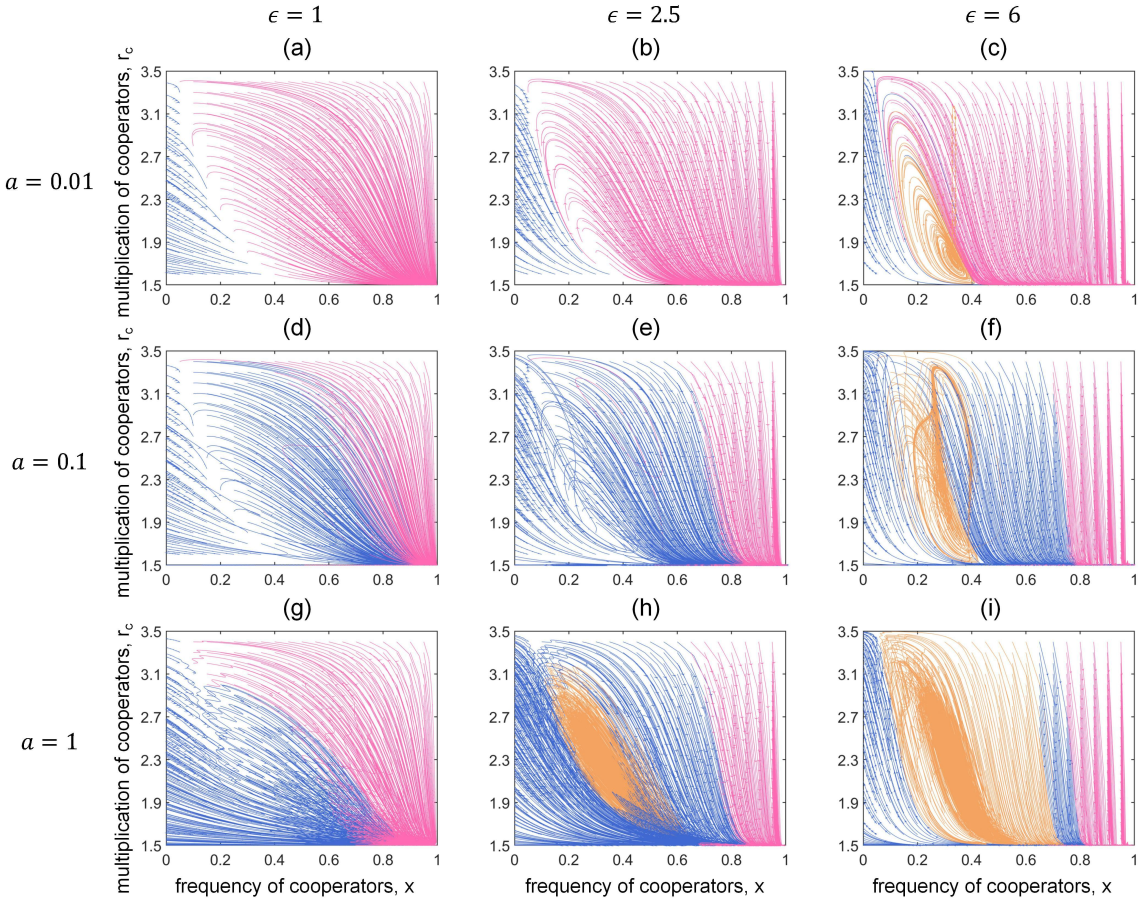

Figure S2.1: Local game-environment evolution in a continuously changing global environment. We use a periodic continuous function, $w_2(t)$, to describe the global environmental fluctuations. Trajectories that eventually evolve to $(x^* = 0)$, $(x^* = 1, r_c^* = \alpha)$ or circulate along an interior closed orbit are distinguished by blue, pink and orange, respectively. Similarly, the cyclic evolution of group cooperation and local environment (orange areas) arises with larger local feedback speeds ($\epsilon = 6$) and faster global environmental fluctuations ($a = 1$). For easy to compare, the values of parameters in all panels are the same as in Fig 4: $N = 4$, $\alpha = 1.5$, $\beta = 3.5$, $\theta = 0.5$, $r_d = 0.6$ and $\delta = -\frac{\pi}{2}$

Fig S2.2i-l present game-environment evolution under discretely changing environment given by $w_4(t)$. Results also show the emergence of cyclic evolution (represented by orange trajectories in Fig S2.2i). We present some representative trajectories by selecting different initial points in Fig S2.2j. Noteworthy, the orange and pink trajectories display the status of the cyclic evolution of group cooperation and the local environment. Take the orange trajectory as an example. The trajectory approaches fixed point 1 (labeled in Fig S2.2j) during the first T time, then seized by fixed point 2 when the global environment changes to the second stage. When the global environment evolves to the third stage, unexpectedly, the dynamical trajectory is not actually captured but is still attracted by fixed point 3, which subsequently is seized by fixed point 2 again. Finally, as fixed point 2 is within the attract domain of fixed point 1, the trajectory forms a complete loop, leading to the cyclic evolution along an inte-

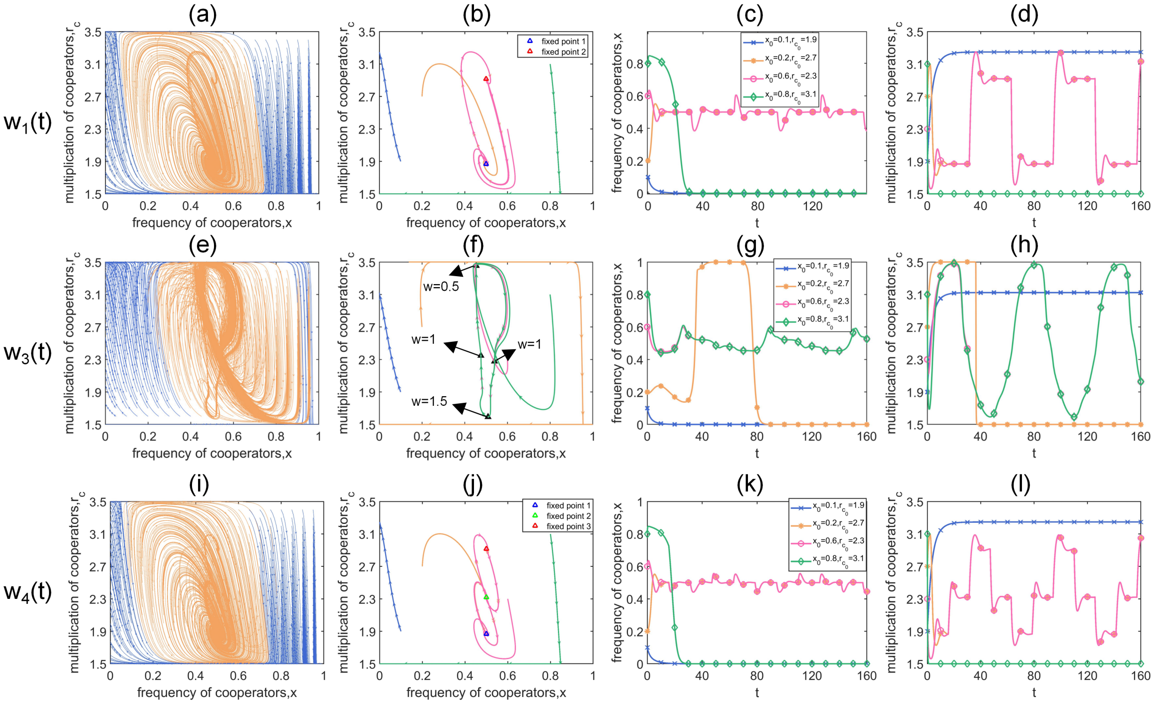

Figure S2.2: Emergence of cyclic evolution of group cooperation and the local environment under periodically changing global environments given by $\boldsymbol{w_3(t)}$. (a) Local game-environment evolution. (b) Four typical dynamic trajectories with different initial conditions. (c-d) Time evolution of x and r_c, corresponding to the colored trajectories in (b). Parameters: $N = 4$,$\alpha = 1.5$, $\beta = 3.5$, $\theta = 1$, $r_d = 1.2$, $a = 0.1$, $\epsilon = 4$.

rior closed orbit consisting of four path segments. The time-evolution pictures (see Fig S2.2k and S2.2l) clearly present the periodic behaviors of x and r_c. This complicated evolutionary process, which shows the important characteristics emerged both in discrete environment $w_1(t)$ where fixed point 1 and fixed point 2 are in the mutual attraction domains and in continuous environment $w_3(t)$ where the dynamical path is not captured yet are strongly attracted by the fixed point, supports our limit thought which explains the formation of the closed orbit under continuously changing environment to some extent.
