# Supplementary material for: Nonlinear eco-evolutionary games with global environmental fluctuations and local environmental feedbacks
Source: PLoS Comput Biol. 2023 Jun 28;19(6):e1011269. doi: 10.1371/journal.pcbi.1011269 (PMC10335700; doi:10.1371/journal.pcbi.1011269)
Supplement: S3 Appendix — We conduct sensitive analysis on all parameters including the multiplication factor of defectors rd, the group size N, the distribution ratio of the expected total payoffs of cooperators and defectors θ, the range of cooperators’ multiplication factor [α, β] and the initial phase δ. (PDF) [file pcbi.1011269.s003.pdf]

S3 Appendix. Sensitive analysis on modeling parameters

In this part, we conduct sensitive analysis on all parameters including the multiplication factor of defectors r_d, the group size N, the distribution ratio of the expected total payoffs of cooperators and defectors θ, the range of cooperators' multiplication factor $[\alpha, \beta]$ and the initial phase δ. We aim to show how these factors affect the emergence of the interior closed orbit in dynamic global environments, which is the most important new finding of our work.

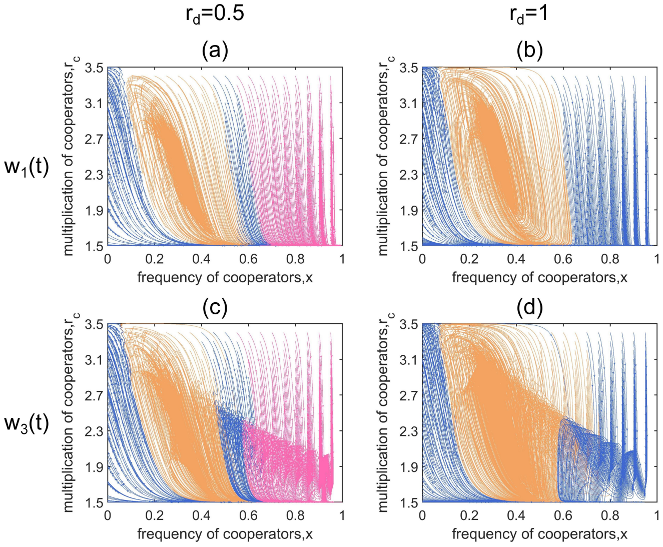

Figure S3.1: Game-environment evolution under different multiplication factors of defector r_d. Trajectories on $x - r_c$ phase plane eventually evolve to $x^* = 0$, $(x^* = 1, r_c^* = \alpha)$ or circulate along an interior closed orbit, which are distinguished by blue, pink and orange, respectively. In all panels, $N = 4$, $\alpha = 1.5$, $\beta = 3.5$, $\theta = 0.5$, $a = 1$, $\epsilon = 6$, $\delta = 0$.

We first study the influence of r_d. Fig S3.1 presents the phase plane of game-environment evolution showing trajectories of different initial points. Specifically, Fig S3.1a and S3.1b describes situations in discretely varying global environment under different r_d, while Fig S3.1c and S3.1d corresponds to continuously changing global environment. In each figure, there are always three kinds of trajectories represented by different colors, where orange lines represent trajectories evolving along an interior closed orbit. It indicates that the emergence of the interior closed orbit is robust on different r_d, whatever the environment is discretely varying or continuously changing. In addition, by comparing Fig S3.1a and S3.1b or Fig S3.1c and S3.1d, we find that the value of r_d indeed affects the range of orange region, i.e., the range of trajectories eventually evolving along the interior closed orbit.

Then we discuss the influence of N in Fig S3.2. We find that the group size N could significantly affect the range of the orange region. Specifically, a larger group size may hinder the emergence of cyclic evolution, making it more difficult to reach the dynamic coexistence of cooperators and defectors both in discretely varying or continuously changing

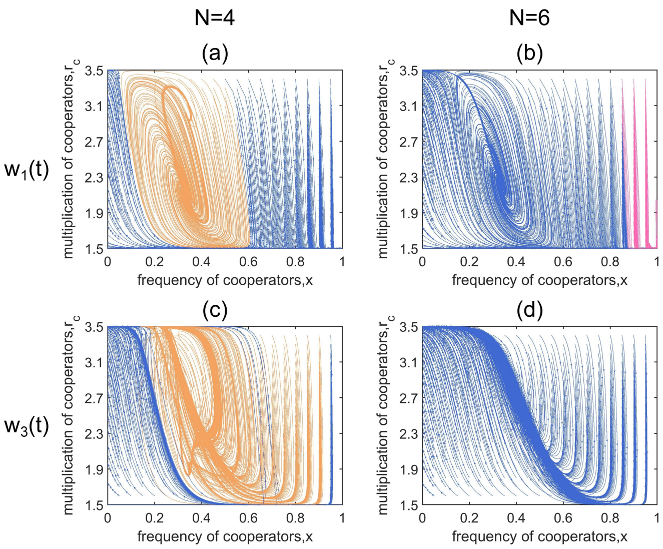

Figure S3.2: Game-environment evolution under different sizes of group N. Trajectories on $x - r_c$ phase plane eventually evolve to $x^* = 0$, $(x^* = 1, r_c^* = \alpha)$ or circulate along an interior closed orbit, which are distinguished by blue, pink and orange, respectively. In all panels, $\alpha = 1.5$, $\beta = 3.5$, $r_d = 1$, $\theta = 0.5$, $a = 0.1$, $\epsilon = 6$, $\delta = 0$.

global environment. This is in line with the simple circumstance revealed by Fig 2(f), where the emergence of a stable interior fixed point requires a larger feedback speed as N becomes larger. It is also noteworthy that a larger N could make full cooperation possible in a discretely varying environment, as shown in Fig S3.2b.

We also explore the influence of θ. Fig S3.3a and S3.3b show that the final position of the interior closed orbit moves as θ changes when the global environment discretely varies, owing to the influence of θ on the fixed points of local game-environment evolution. Fig S3.3c and S3.3d show that the phenomenon of cyclic evolution may disappear with larger θ when the global environment continuously changes.

In addition, we study the influence of α and β. We set $\alpha = 1.5$, $\beta = 3.5$ for Fig S3.4a and S3.4c and $\alpha = 1$, $\beta = 5$ for Fig S3.4b and S3.4d. Apparently, large range of α and β could significantly promote the emergence of dynamic coexistence of cooperators and defectors.

Last, we explore the influence of δ, the initial phase of the function $w_2(t)$ representing the continuously changing environment. Figure S3.5 presents $x - r_c$ phase plane under different δ and changing speed of environment a. Results show that all subfigures have a large range of orange trajectories eventually evolving along an interior closed orbit, although the shapes of the orange range are distinguished in different figures. This indicates that the interior closed orbit always emerges for a large range of δ and that the value of δ can alter the attraction range of the interior closed orbit.

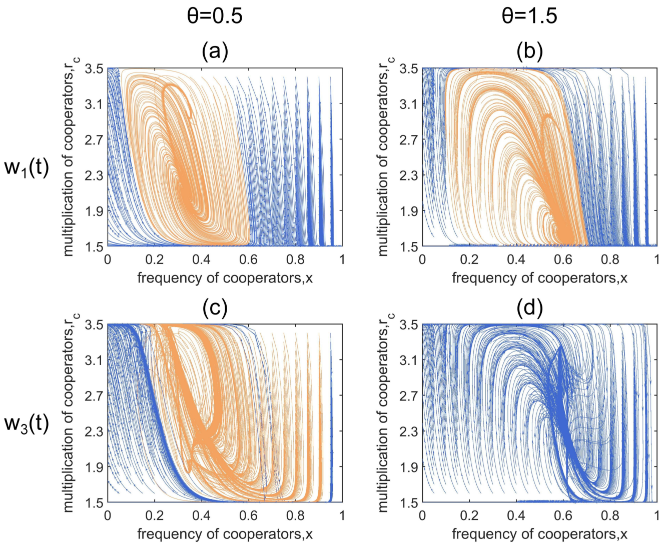

Figure S3.3: Game-environment evolution under different distribution ratio of the expected total payoffs of the cooperators and defectors $\boldsymbol{\theta}$. Trajectories on $x-r_c$ phase plane eventually evolve to $x^*=0$, $(x^*=1, r_c^*=\alpha)$ or circulate along an interior closed orbit, which are distinguished by blue, pink and orange, respectively. In all panels, $N=4$, $\alpha=1.5$, $\beta=3.5$, $r_d=1$, $a=0.1$, $\epsilon=6$, $\delta=0$.

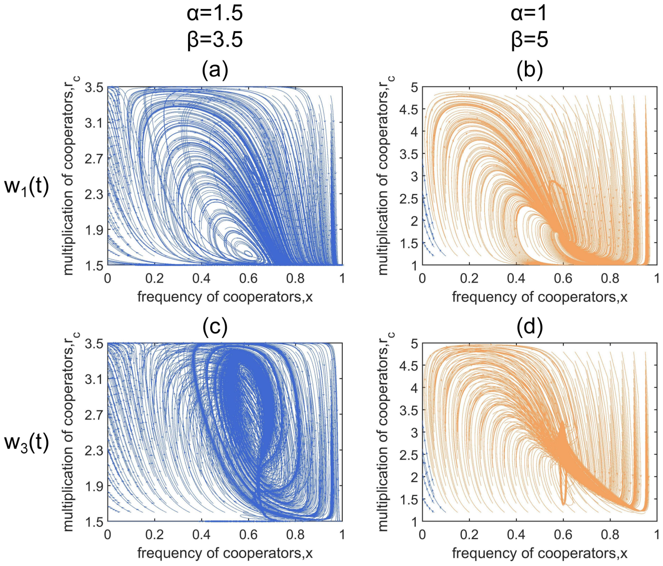

Figure S3.4: Game-environment evolution under different cooperators' multiplication factor $[\boldsymbol{\alpha}, \boldsymbol{\beta}]$. Trajectories on $x-r_c$ phase plane eventually evolve to $x^*=0$, $(x^*=1, r_c^*=\alpha)$ or circulate along an interior closed orbit, which are distinguished by blue, pink and orange, respectively. In all panels, $N=4$, $r_d=1$, $\theta=1.5$, $a=0.1$, $\epsilon=2$, $\delta=0$.

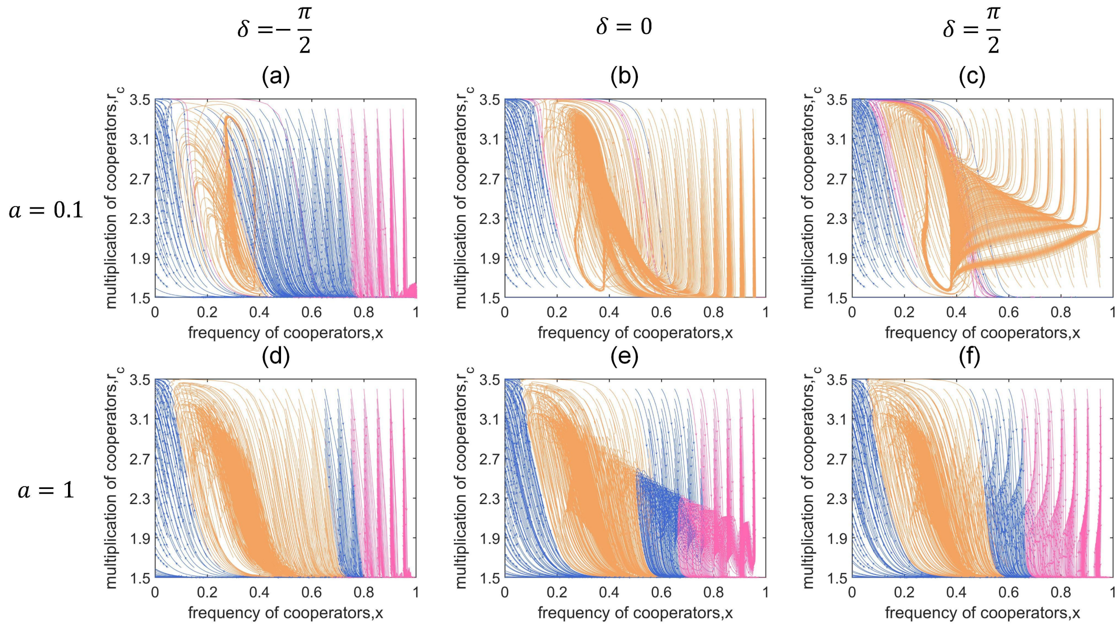

Figure S3.5: Game-environment evolution under different initial phase δ and self-changing speed a of continuously changing global environment. In $x - r_c$ plane, there are always three kinds of trajectories: the blue, pink and orange trajectories will eventually evolve to $x^* = 0$, $(x^* = 1, r_c^* = \alpha)$ and circulate along a closed orbit, respectively. In all panels, $N = 4$, $\alpha = 1.5$, $\beta = 3.5$, $\theta = 0.5$, $r_d = 0.6$, $\epsilon = 7$.
