# Supplementary material for: Nonlinear eco-evolutionary games with global environmental fluctuations and local environmental feedbacks
Source: PLoS Comput Biol. 2023 Jun 28;19(6):e1011269. doi: 10.1371/journal.pcbi.1011269 (PMC10335700; doi:10.1371/journal.pcbi.1011269)
Supplement: Attachment — Submitted filename: report update (Reviewer 2).pdf [file pcbi.1011269.s004.pdf]

In this paper, the authors explore the effect of the interplay between local and global environmental fluctuations on the evolution of cooperation in a n-player public goods game. Here, local environmental fluctuations are encoded as changes in the productivity factor for cooperators, while global fluctuations determine whether cooperation is promoted or discounted by the environment. In agreement with previous studies, the authors recover periodic behavior of the system in a periodic environment. Interestingly, the periodic solution for chosen parameter values fluctuates in the interior of the (0,1)-interval, while hardly ever going close to either 0 or 1. Another interesting observation is that the system seems to have a continuum of attractors for each value of the environmental functions and once these parameters evolve with time, the system behavior follows these attractors, which is in turn determined by the differences in timescales. Overall, I enjoyed reading this manuscript, but I have a few comments to the authors:

1. As my main concern, I found it hard to compare results for different model variations due to very different choice of parameter values. It would be nice if the authors could provide some form of sensitivity analysis (could also be just numerical) for all parameters (including N, theta, alpha and beta). In addition, it would be nice if the authors provide motivation for the parameter values they choose. For example, why r_d<alpha? Why these choices differ for different model versions? I could only find an explanation for these changes for epsilon. Why w_1 is changing between 1.2 and 0.8 and w_2 between 0.5 and 1.5?
2. The authors are trying to make connections to human societal issues. While I appreciate these comparisons, I believe that more biological connections are needed for the readers of this journal. Examples of parts of the paper where I would look for a better-fitting biological illustration are: lines 36-41, extra example in line 45, lines 63-68, 126-129, 143-144, 285-286, 337-338, 340-343. One possibility is to look for these examples in the microbial/systems biology literature. For example, cancer cells or complex bacterial communities.
3. The authors consider two functional forms of w(t), which are in principle very similar (and induce similar behavior). While I understand that the model is already quite complex, I wonder how hard would it be to analyze an example when w(t) also depends on x? Could it be that the global environmental conditions are also dependent on the frequency of cooperators, for example, in a threshold-function manner?
4. As a general comment about figures, I found the text in figures too small making it hard to read. The authors might save some space by placing the panels closer to each other and naming axis only once per row/column in the figure. In addition, while I do not have strong feelings about it, it might be worth separating axis in fig. 2(b) as frequency x runs only up to 1. It might also be worth it to increase the thickness of lines whenever possible.
5. Generally, I think that the paper needs some proof-reading. I provide a list of typos/corrections, but please note that it is not exhaustive:
    - Line 14: ~~the~~ heterogeneity of players
    - Line 19: Accordingly might be not the right word here
    - Line 33: a wide range
    - Line 35: “and etc” seems a bit too casual
    - Line 43: socio-ecological (this comment also applies to all other instances of socio-something)
    - Line 179: in a skilled project,

- Line 258: evolution (singular, similar to other instances in the manuscript)
- Line 297: on the other hand
- Line 305: furthermore might be not the best word here
- Line 314: ~~the~~ cooperators
- Line 321: global dynamic environments?
- To beautify the numbers a bit, can the authors try to find rational numbers representing the special values of parameters? Say, 0.4444 can be written as 4/9
