# Supplementary material for: Nonlinear eco-evolutionary games with global environmental fluctuations and local environmental feedbacks
Source: PLoS Comput Biol. 2023 Jun 28;19(6):e1011269. doi: 10.1371/journal.pcbi.1011269 (PMC10335700; doi:10.1371/journal.pcbi.1011269)
Supplement: Attachment — Submitted filename: renamed_c8135.pdf [file pcbi.1011269.s005.pdf]

Point-by-point response to the reviewers' comments

Manuscript PCOMPBIOL-S-22-02376

Reviewer #1:

The authors in this paper combine global environmental fluctuations and local environmental feedbacks in a human-environment system. They consider individuals playing a public goods game where more than one cooperator is present in the interactions. The global fluctuations can be depicted by introducing the synergy and discounting of cooperation (a nonlinear factor omega). On the other hand, the local feedbacks can be captured by coupling the individual behavior and the local game environment (how the multiplication factor r change in the public goods game for cooperators).

Given that everything takes place in a well-mixed, infinitely large population, the system can be described by a pair of ordinary different equations mathematically. The authors study static and dynamic global environments (that is, whether and how omega changes) and obtain some very interesting evolutionary outcomes including the emergence of cyclic evolutions. The findings are supported with both theoretical analysis and numerical approximations (I see no agent-based simulations).

Their work shed light on how diverse dynamics may emerge due to nonlinear interactions between individual performance and environment fluctuations. Nevertheless, I have a few conceptual and mathematical questions that I hope the authors could answer.

We thank the reviewer for these thoughtful criticisms and constructive suggestions. We have considered each suggestion carefully and have now provided more details about our results to improve the clarity and rigour of the manuscript. The detailed responses (in blue) are as follows and the corresponding changes in the manuscript are also quoted (in red) for convenience.

(1) The group size N of the public goods game appears in equation (10). How would this parameter affect the evolution of cooperation? (as there is no results from agent-based simulations, at least the authors can do it theoretically/numerically?)

We thank the reviewer for this suggestion. We have supplemented detailed results on the influence of group size N numerically in Fig. 2 and Fig. 8. When the global environment is static, we show complicated coupled influence of N and other parameters on the equilibrium states of local game-environment evolution revealed by r_c^* and ϵ^*, respectively (Fig 2(e)(f)). Further, we find that a larger N may destroy the cyclic evolution of cooperators and defectors in dynamic global environments (Figure S2). The corresponding changes in the manuscript are as following：

Page7(Fig 2)

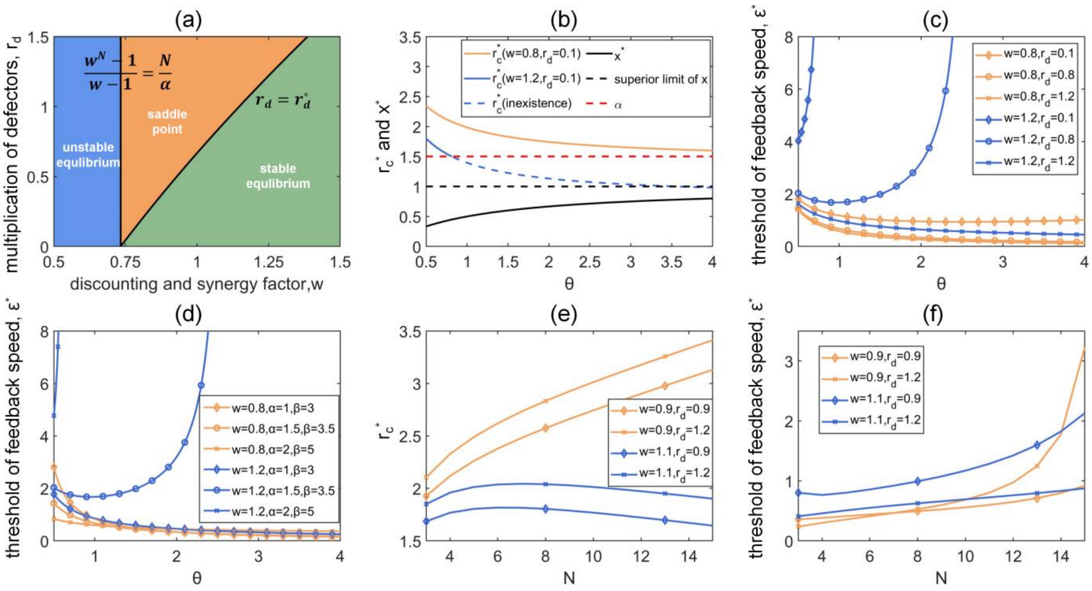

Fig 2. Effects of varying modeling parameters on equilibrium states of local game-environment evolution in static global environments. Panel (a) shows the combined influence of w and r_d on the stability of the boundary fixed point (1, α). Panel (b) presents the trends of the interior fixed point (x^*, r_c^*) as θ changes (the dashed blue line indicates that the value of r_c^* is out of parameter range [α, β]). Panel (c) and (d) show how the threshold of the relative feedback speed ϵ^*, which is the minimum value leading to the interior stability, is affected by different parameters. Panel (e) and (f) show the detailed influence of group size N on the equilibrium states revealed by r_c^* and ϵ^*, respectively. Parameters: $N = 4$ in (a-d), $\alpha = 1.5$, $\beta = 3.5$ in (a-c, e-f) and $r_d = 0.8$ in (d).

Page8(Results)

Similar trends can also be observed under different combinations of α and β (Fig 2(d)).

Furthermore, we show how the group size N influences the equilibrium states of local game-environment evolution revealed by r_c^* and ϵ^*, respectively (Fig 2(e) and (f)). To ensure that the interior fixed point always exists as N changes, we set $w = 0.9$ and 1.1, $r_d = 0.9$ and 1.2, $\theta = 2$. We find that in a discounting global environment, r_c^* is approximately in direct proportion to N, while in the synergy condition, r_c^* increases first and then decreases gradually as N increases (Fig 2(e)). Interestingly, r_c^* is always larger when w is smaller or r_d is larger regardless the changes in N. This is intuitive: when the global environment is worse or the multiplication factor of defectors is larger, the payoff coefficient of cooperators should be increased in order to maintain the stable coexistence of cooperators and defectors. We also find that ϵ^* increases as N increases under all four sets of parameters, indicating that the emergence of the stable interior fixed point requires faster local environmental feedback when the group size becomes larger (Fig 2(f)).

Page11(Results)

In addition, we confirm that the emergence of cyclic evolution is robust on different other parameters, including the multiplication factor of defectors r_d, the distribution ratio of the

expected total payoffs of cooperators and defectors θ, the range of cooperators' multiplication factor [α, β] and the initial phase δ (see S2 Appendix). It is noteworthy that these factors have strong and complicated impacts on the range of initial values that could eventually stabilize to full cooperation (x^*= 1 and r_c^* = α) or to the interior closed orbit. We also find that a larger group size N may hinder the dynamic coexistence of cooperators and defectors (see S2 Appendix. Fig S2).

Supporting Information(S2 Appendix)

In this part, we conduct sensitive analysis on all parameters including the multiplication factor of defectors r_d, the group size N, the distribution ratio of the expected total payoffs of cooperators and defectors θ, the range of cooperators' multiplication factor [α, β] and the initial phase δ. We aim to show how these factors affect the emergence of the interior closed orbit in dynamic global environments, which is the most important new finding of our work.

Supporting Information(S2 Appendix)

Then we discuss the influence of N in Figure S2. We find that the group size N could significantly affect the range of orange region. Specifically, a larger group size may hinder the emergence of cyclic evolution, making it more difficult to reach to the dynamic coexistence of cooperators and defectors both in discretely varying or continuously changing global environment. This is in line with the simple circumstance revealed by Fig 2(f), where the emergence of stable interior fixed point requires a larger feedback speed as N becomes larger. It is also noteworthy that a larger N could make full cooperation possible in a discretely varying environment, as shown in Figure S2(b).

Supporting Information(Figure S2)

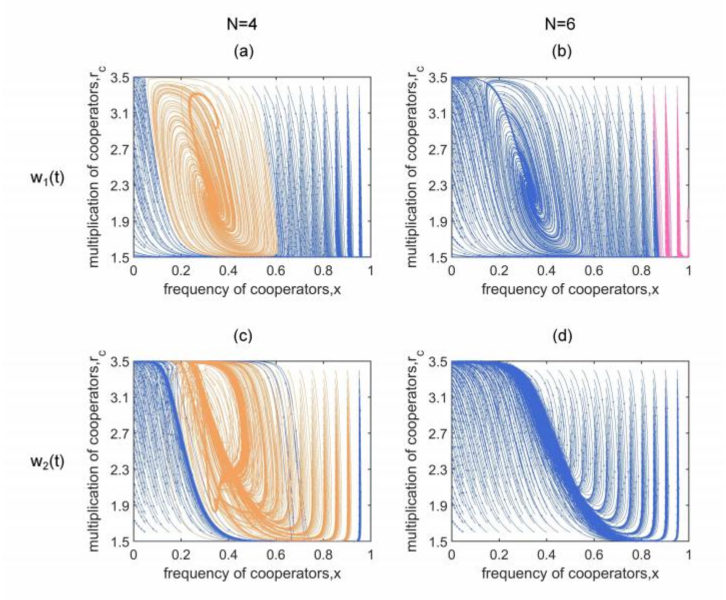

Figure S2. Game-environment evolution under different group size N. Trajectories on x-r_c phase plane eventually evolve to x^* = 0, (x^* = 1, r_c^* = α) or circulate along an interior closed orbit, which are distinguished by blue, pink and orange, respectively. In all panels, α = 1.5, β = 3.5, r_d = 1, θ = 0.5, a = 0.1, ϵ = 6, δ = 0.

(2) Are there any real-world examples from biology/economy/social science/… that resonate with the findings in the paper? I am particularly interested. After all, the analysis from a dynamical-system perspective may not be intuitive enough.

We thank the reviewer for this constructive comment which is a great complement to the significance of our work. We have added some real-word examples from social science, biology and economy in the Results part and Conclusion part.

New References

37. Li Y, Wang ZB, Zhang XY, Dang YR, Sun LL, Zhang WP, et al. Experimental evidence for long-term coexistence of copiotrophic and oligotrophic bacteria in pelagic surface seawater. Environmental Microbiology. 2021;23(2):1162–1173

62. Sanchez A, Gore J. Feedback between population and evolutionary dynamics determines the fate of social microbial populations. PLoS biology. 2013;11(4):e1001547.

64. Babajanyan S, Koonin EV, Cheong KH. Can Environmental Manipulation Help Suppress Cancer? Non-Linear Competition Among Tumor Cells in Periodically Changing Conditions. Advanced Science. 2020;7(16):2000340

67. Weitz JS, Park SW, Eksin C, Dushoff J. Awareness-driven behavior changes can shift the shape of epidemics away from peaks and toward plateaus, shoulders, and oscillations. Proceedings of the National Academy of Sciences. 2020;117(51):32764–32771.

68. Merow C, Urban MC. Seasonality and uncertainty in global COVID-19 growth rates. Proceedings of the National Academy of Sciences. 2020;117(44):27456–27464.

69. Bergman A, Sella Y, Agre P, Casadevall A. Oscillations in US COVID-19 incidence and mortality data reflect diagnostic and reporting factors. Msystems. 2020;5(4):e00544–20.

70. Glaubitz A, Fu F. Oscillatory dynamics in the dilemma of social distancing. Proceedings of the Royal Society A. 2020;476(2243):20200686

73. Bowles S, Choi JK, Hopfensitz A. The co-evolution of individual behaviors and social institutions. Journal of theoretical biology. 2003;223(2):135–147.

Page12-13(Results)

Such periodic evolution is actually in line with the intuitions from real world in a way that the group cooperation will neither disappear completely nor always be maintained at the highest level in many complex systems, for instance, the seasonal oscillating dynamics of the COVID-19 infection [67-70], the dynamic adjustment of big companies in the economic cycles and the oscillating abundance of bacteria and cancer cells in periodically varying environments [37, 64].

Page14-15(Discussion)

To sum up, our model provides profound insight into how diverse group behaviors, especially oscillating convergence and cyclic evolution, can emerge from the nonlinear interactions between strategies and dynamic environments. Our work helps understand the complexity of social dilemmas in an ever-changing world. For instance, the game dynamics of the Labor and Capital in

social-economic system can be easily translated into our frame: The companies engaging staff can be mapped into the local environment, the state of which coevolves with individual strategies [73]. Outside, the trends of the corresponding industry or the economic state of the country, which can largely determine the marginal benefits of strategic behaviors, can be modeled as the global environment. Such an approach allows us to examine how the evolutionary stable state of the labor-capital game is jointly influenced by factors such as the incentive mechanisms of the company and the development of the economies. Our results also have important practical value in systems biology. Studies have shown that the global periodically varying environment associated with biotic (blood circulation, nutrients supply) or abiotic (periodical drug usage) variations could induce nonlinear competition between different types of tumor cells in local environments, which drastically changes the evolutionary outcomes and results in oscillating dynamics of tumor population, breaking the dominance of a certain type of tumor cell [64]. Our model incorporating nonlinear local interactions as well as general mechanisms of global environmental changes thus paves ways for a more confined understanding on such dynamical processes of cancer evolution. For instance, the parameters in global environmental changes could be exploited for designing milder control strategies on tumor growth. Similarly, in microbial system, the concentration of casein, corresponding to the global environment, could lead to an oscillation coexistence of copiotroph (W04) and oligotroph (Y09) in the local environment [37]. In microbial experiments, the dilution factor in the growth medium significantly influences the local feedback loop between laboratory yeast population and the dynamics of cooperators, the SUC2 gene [62]. Our model can be used to describe these complex microbial systems and may help in establishing effective control methods that could tune the system evolution to a desired direction.

(3) The captions of the figures can be more self-contained (especially Figure 3-6).

We thank the reviewer for the helpful suggestion. We have updated the captions of figures by adding detailed explanation. We present the new captions below.

Page7(Fig 2)

Fig 2. Effects of varying modeling parameters on equilibrium states of local game-environment evolution in static global environments. Panel (a) shows the combined influence of w and r_d on the stability of the boundary fixed point $(1, \alpha)$. Panel (b) presents the trends of the interior fixed point (x^*, r_c^*) as θ changes (the dashed blue line indicates that the value of r_c^* is out of parameter range $[\alpha, \beta]$). Panel (c) and (d) show how the threshold of the relative feedback speed ϵ^*, which is the minimum value leading to the interior stability, is affected by different parameters. Panel (e) and (f) show the detailed influence of group size N on the equilibrium states revealed by r_c^* and ϵ^*, respectively. Parameters: $N = 4$ in (a-d), $\alpha = 1.5, \beta = 3.5$ in (a-c, e-f) and $r_d = 0.8$ in (d).

Page9(Fig 3)

Fig 3. Phase plane dynamics of local game-environment (x-r_c) evolution with $N = 4$, $\alpha = 1.5$, $\beta = 3.5$, $\theta = 1.5$, $r_d = 1$. We set $w = 0.8, 1, 1.2$ to describe the scenarios of discounting, linear, and synergy PGG in each column, and the corresponding ϵ^* are 0.29, 0.44, 1.06, respectively. We therefore choose $\epsilon = 0.09, 0.24, 0.86$ for the first row such that $\epsilon < \epsilon^*$, and $\epsilon = 0.79, 0.94, 1.56$ for the third row such that $\epsilon > \epsilon^*$. The blue, pink and gray areas represent the basin of attraction of different fixed points ($x^* = 0$), ($x^* = 1$, $r_c^* = \alpha$) and ($x^* = \frac{\theta}{\theta+1}, r_c^* =$

$\frac{N(w-1)+r_d((wx^*-x^*+1)^{N-1}-1)}{w(wx^*-x^*+1)^{N-1}-1}$), respectively. The stable coexistence of cooperators and defectors only occurs when $\epsilon > \epsilon^*$ (the third row), as predicted theoretically, while a synergistically enhanced global environment significantly promotes the emergence of full cooperation (the third column).

Page11(Fig 4)

Fig 4. Local game-environment evolution in a discretely varying global environment. We use a periodic piecewise function, $w_1(t)$, to describe the global environmental fluctuations. We uniformly select initial points on the x-r_c plane and plot the corresponding dynamical trajectories by numerically solving Eq. 9. Trajectories that eventually evolve to (x^*= 0), (x^*= 1, r_c^* = α) or circulate along an interior closed orbit are distinguished by blue, pink and orange, respectively. The emergence of cyclic evolution of group cooperation and local environment (orange areas), which cannot be observed in static global environments, indicates that the global environmental fluctuations could fundamentally alter the evolutionary outcomes in local feedback-evolving game. In all panels, N = 4, α = 1.5, β = 3.5, θ = 0.5, r_d = 0.6.

Page12(Fig 5)

Fig 5. Local game-environment evolution in a continuously changing global environment. We use a periodic continuous function, $w_2(t)$, to describe the global environmental fluctuations. Trajectories that eventually evolve to (x^*= 0), (x^*= 1, r_c^* = α) or circulate along an interior closed orbit are distinguished by blue, pink and orange, respectively. Similarly, the cyclic evolution of group cooperation and local environment (orange areas) arises with larger local feedback speeds ($\epsilon = 6$) and faster global environmental fluctuations (a=1). For easy to compare, the values of parameters in all panels are the same as in Fig 4: N = 4, α = 1.5, β = 3.5, θ = 0.5, r_d = 0.6 and $\delta = -\frac{\pi}{2}$.

Page13(Fig 6)

Fig 6. Emergence of cyclic evolution of group cooperation and local environment under periodically changing global environments given by $w_1(t)$ and $w_2(t)$. The first column shows local game-environment evolution similar to Fig 4 and Fig 5. The second column displays four typical dynamic trajectories in detail, particularly the interior closed yet irregular orbits. The last two columns present time evolution of the frequency of cooperators x and the multiplication factor of cooperators r_c under different initial conditions, corresponding to the colored trajectories in the second column. Results show that the formation of cyclic evolution under discrete global environment is due to the fact that the two fixed points are in the mutual attraction domains (b-d), while in continuous situation such phenomena could be interpreted using limit thought (f-h). Parameters: N = 4, α = 1.5, β = 3.5, θ = 1, r_d = 1.2, a = 0.1, ϵ = 4, δ = 0.

There are also some typos in the manuscript. The authors may do a more careful proofread. To name a few:

Page 4, line 101: an extra ‘the’.

Thank you, we have deleted the extra ‘the’.

Page 10, line 249-250: ‘max’ and ‘min’ should not be italic.

Thank you, we have corrected the text accordingly.

Besides, I cannot open the URL in Data and Code Availability.

We thank the reviewer for the reminder and we have now made our code available at https://github.com/lileason/PCOMPBIOL-D-22-01825.git

Above all, it is a novel paper and adds to the literature of eco-evolutionary games. I would suggest acceptance with a minor revision.

We hope that the modifications will meet with approval. Once again, thank you very much for the professional comments and suggestions on our manuscript.

Reviewer #2:

In this paper, the authors explore the effect of the interplay between local and global environmental fluctuations on the evolution of cooperation in a n-player public goods game. Here, local environmental fluctuations are encoded as changes in the productivity factor for cooperators, while global fluctuations determine whether cooperation is promoted or discounted by the environment. In agreement with previous studies, the authors recover periodic behavior of the system in a periodic environment. Interestingly, the periodic solution for chosen parameter values fluctuates in the interior of the (0,1)-interval, while hardly ever going close to either 0 or 1. Another interesting observation is that the system seems to have a continuum of attractors for each value of the environmental functions and once these parameters evolve with time, the system behavior follows these attractors, which is in turn determined by the differences in timescales. Overall, I enjoyed reading this manuscript, but I have a few comments to the authors (please see the report attached).

We thank the reviewer for the positive feedback and instructive criticisms. We have incorporated all your insights and suggestions to the revised manuscript and we believe the manuscript has been greatly improved. The detailed responses (in blue) are as follows and the corresponding changes in the manuscript are also quoted (in red) for convenience.

1. As my main concern, I found it hard to compare results for different model variations due to very different choice of parameter values. It would be nice if the authors could provide some form of sensitivity analysis (could also be just numerical) for all parameters (including N, theta, alpha and beta). In addition, it would be nice if the authors provide motivation for the parameter values they choose. For example, why r_d<alpha? Why these choices differ for different model versions? I could only find an explanation for these changes for epsilon. Why w_1 is changing between 1.2 and 0.8 and w_2 between 0.5 and 1.5?

We very much appreciate your constructive comments. Firstly, we have added detailed sensitivity analyses for all parameters in our manuscript. Under static global environment, we added Fig 2(d) to study the influence of α and β on ϵ^*, and Fig 2(e)(f) to discuss the influence of N on r_c^* and ϵ^*. In dynamic global environment, we added Figure S2-S4 in S2 Appendix to show how N, θ, α and β affect the emergence of the interior closed orbit, which is the most important new finding of our work. Secondly, we explained why $r_d \leq \alpha$ in the modeling part. Briefly, we set $r_d \leq r_c$ to describe the widespread phenomenon in microbial and social systems that cooperators have preferential access to the common good, therefore we assume $r_d \leq \alpha \leq r_c$ for the sake of simplicity. Thirdly, for comparison, we choose the same set of parameters and replotted Fig 4 and Fig 5. Lastly, we have modified the range of $w_1(t)$ such that the expectations of synergistically enhanced or discounted influence exerted by $w_1(t)$ and $w_2(t)$ in time interval T are equal. As a result, we set $w_1(t)$ change between $\frac{1}{T}\int_0^{\mathrm{T}}(1-0.5\sin at)\mathrm{d}t \approx 0.7$ and $\frac{1}{T}\int_{\mathrm{T}}^{2\mathrm{T}}(1-0.5\sin at)\mathrm{d}t \approx 1.3$. The detailed updates in the manuscript are as follows.

Sensitivity analysis for all parameters:

Page7(Fig 2)

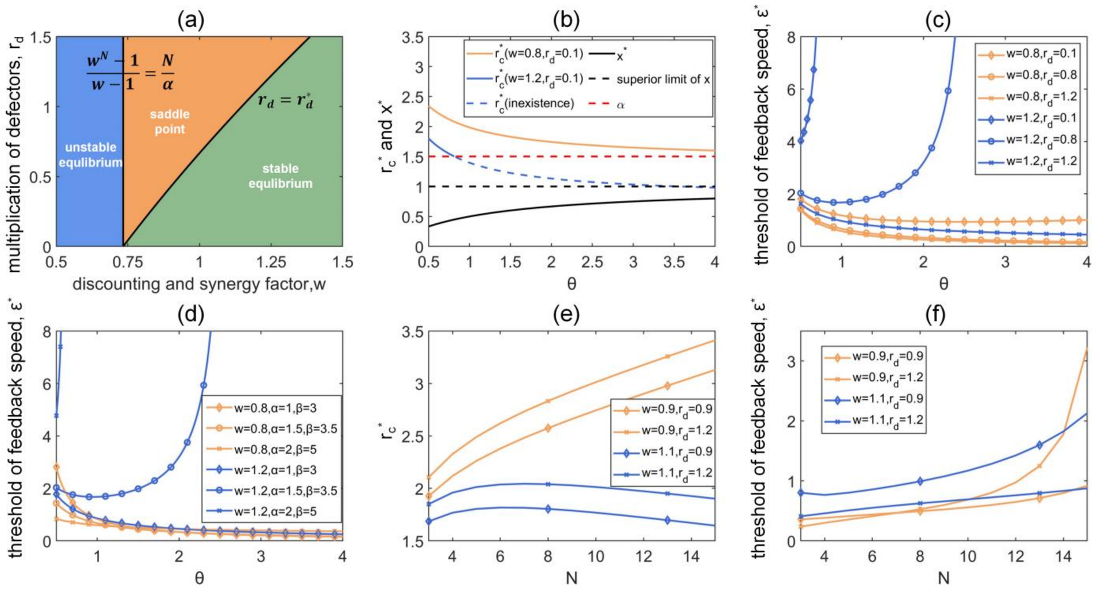

Fig 2. Effects of varying modeling parameters on equilibrium states of local game-environment evolution in static global environments. Panel (a) shows the combined influence of w and r_d on the stability of the boundary fixed point (1, α). Panel (b) presents the trends of the interior fixed point (x^*, r_c^*) as θ changes (the dashed blue line indicates that the value of r_c^* is out of parameter range [α, β]). Panel (c) and (d) show how the threshold of the relative feedback speed ϵ^*, which is the minimum value leading to the interior stability, is affected by different parameters. Panel (e) and (f) show the detailed influence of group size N on the equilibrium states revealed by r_c^* and ϵ^*, respectively. Parameters: $N = 4$ in (a-d), $\alpha = 1.5$, $\beta = 3.5$ in (a-c, e-f) and $r_d = 0.8$ in (d).

Page8(Results)

Similar trends can also be observed under different combinations of α and β (Fig 2(d)).

Furthermore, we show how the group size N influences the equilibrium states of local game-environment evolution revealed by r_c^* and ϵ^*, respectively (Fig 2(e) and (f)). To ensure that the interior fixed point always exists as N changes, we set $w = 0.9$ and 1.1, $r_d = 0.9$ and 1.2, $\theta = 2$. We find that in a discounting global environment, r_c^* is approximately in direct proportion to N, while in the synergy condition, r_c^* increases first and then decreases gradually as N increases (Fig 2(e)). Interestingly, r_c^* is always larger when w is smaller or r_d is larger regardless the changes in N. This is intuitive: when the global environment is worse or the multiplication factor of defectors is larger, the payoff coefficient of cooperators should be increased in order to maintain the stable coexistence of cooperators and defectors. We also find that ϵ^* increases as N increases under all four sets of parameters, indicating that the emergence of the stable interior fixed point requires faster local environmental feedback when the group size becomes larger (Fig 2(f)).

Page11(Results)

In addition, we confirm that the emergence of cyclic evolution is robust on different other parameters, including the multiplication factor of defectors r_d, the distribution ratio of the expected total payoffs of cooperators and defectors θ, the range of cooperator's multiplication factor $[\alpha, \beta]$ and the initial phase δ (see S2 Appendix). It is noteworthy that these factors have strong and complicated impacts on the range of initial values that could eventually stabilize to full cooperation (x^*= 1 and $r_c^* = \alpha$) or to the interior closed orbit. We also find that a larger group size N may hinder the dynamic coexistence of cooperators and defectors (see S2 Appendix. Fig S2).

Supporting Information(S2 Appendix)

In this part, we conduct sensitive analysis on all parameters including the multiplication factor of defectors r_d, the group size N, the distribution ratio of the expected total payoffs of cooperators and defectors θ, the range of cooperator's multiplication factor $[\alpha, \beta]$ and the initial phase δ. We aim to show how these factors affect the emergence of the interior closed orbit in dynamic global environments, which is the most important new finding of our work.

Supporting Information(S2 Appendix)

Then we discuss the influence of N in Figure S2. We find that the group size N could significantly affect the range of orange region. Specifically, a larger group size may hinder the emergence of cyclic evolution, making it more difficult to reach to the dynamic coexistence of cooperators and defectors both in discretely varying or continuously changing global environment. This is in line with the simple circumstance revealed by Fig 2(f), where the emergence of stable interior fixed point requires a larger feedback speed as N becomes larger. It's also noteworthy that a larger N could make full cooperation possible in a discretely varying environment, as shown in Figure S2(b).

Supporting Information(S2 Appendix)

We also explore the influence of θ. Figure S3(a)(b) show that the final position of the interior closed orbit moves as θ changes when the global environment discretely varies, owing to the influence of θ on the fixed points of local game-environment evolution. Figure S3(c)(d) show that the phenomenon of cyclic evolution may disappear with larger θ when the global environment continuously changes.

Supporting Information(S2 Appendix)

In addition, we study the influence of α and β. We set α=1.5, β=3.5 for Figure S4(a)(c) and α = 1, β = 5 for Figure S4(b)(d). Apparently, large range of α and β could significantly promote the emergence of dynamic coexistence of cooperators and defectors.

Supporting Information(Figure S2)

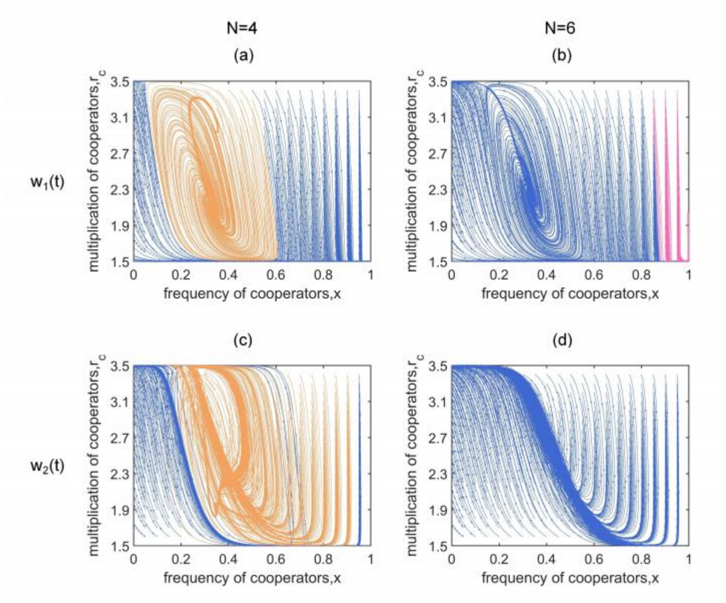

Figure S2. Game-environment evolution under different sizes of group N. Trajectories on x-r_c phase plane eventually evolve to $x^*=0$, ($x^*=1$, $r_c^*=\alpha$) or circulate along an interior closed orbit, which are distinguished by blue, pink and orange, respectively. In all panels, $\alpha=1.5$, $\beta=3.5$, $r_d=1$, $\theta=0.5$, $a=0.1$, $\epsilon=6$, $\delta=0$.

Supporting Information(Figure S3)

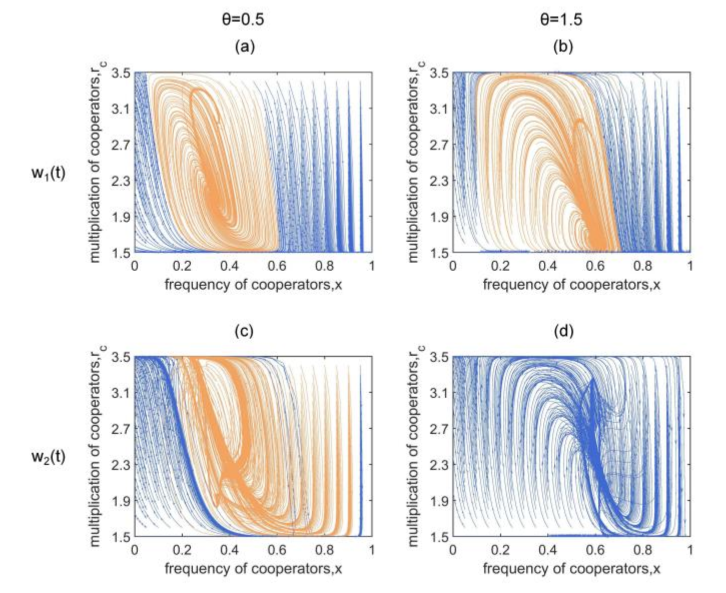

Figure S3. Game-environment evolution under different distribution ratio of the expected total payoffs of cooperators and defectors θ. Trajectories on x-r_c phase plane eventually evolve to $x^*=0$, ($x^*=1$, $r_c^*=\alpha$) or circulate along an interior closed orbit, which are distinguished by blue, pink and orange, respectively. In all panels, $N=4$, $\alpha=1.5$, $\beta=3.5$, $r_d=1$, $a=0.1$, $\epsilon=6$, $\delta=0$.

Supporting Information(Figure S4)

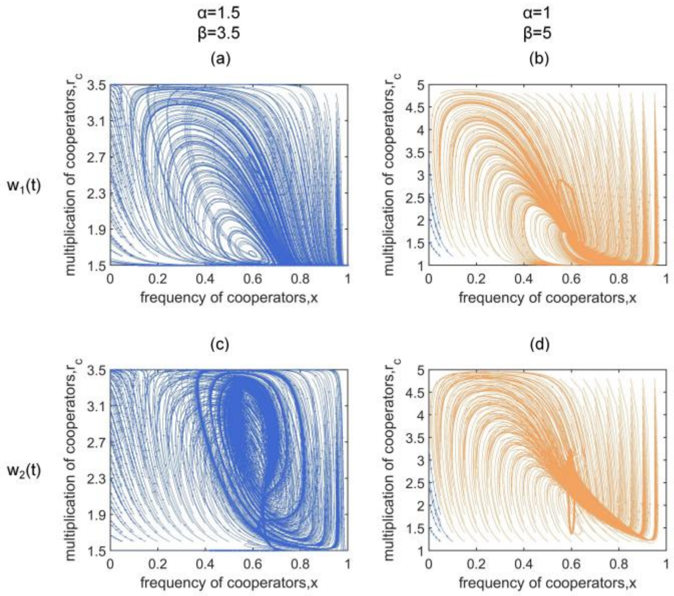

Figure S4. Game-environment evolution under different range of cooperator's multiplication factor $[\alpha, \beta]$. Trajectories on x-r_c phase plane eventually evolve to $x^*=0$, ($x^*=1$, $r_c^*=\alpha$) or circulate along an interior closed orbit, which are distinguished by blue, pink and orange, respectively. In all panels, $N=4$, $r_d=1$, $\theta=1.5$, $a=0.1$, $\epsilon=2$, $\delta=0$.

Why r_d<alpha:

Page6(Methods)

Besides, to reduce model complexity, the defectors' multiplication factor r_d is set to be constant. We assume $r_d \leqslant r_c$ to describe the widespread phenomenon that cooperators have preferential access to the common good in microbial and social systems [56,57,65,66]. For the sake of simplicity, we set $r_d \leqslant \alpha \leqslant r_c$ throughout the paper.

Why these choices differ for different model versions:

Page11(Fig 4)

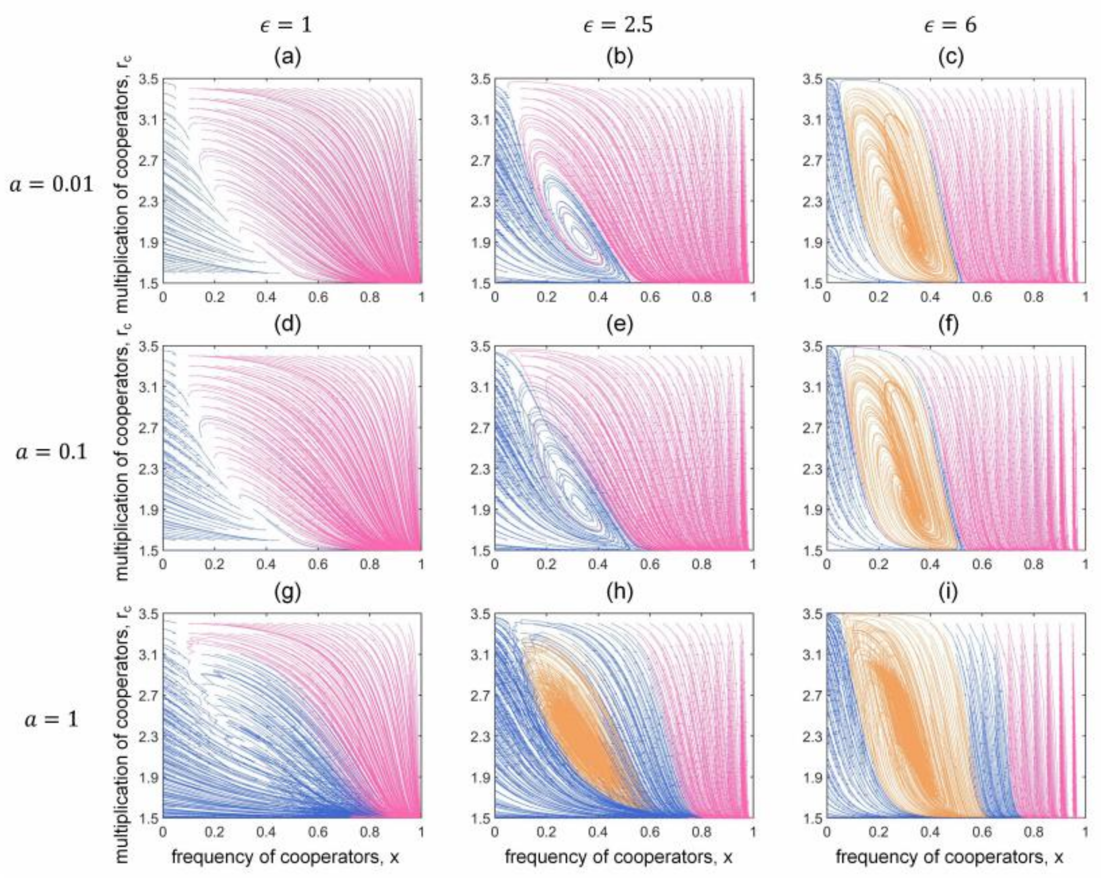

Fig 4. Local game-environment evolution in a discretely varying global environment. We use a periodic piecewise function, $w_1(t)$, to describe the global environmental fluctuations. We uniformly select initial points on the x-r_c plane and plot the corresponding dynamical trajectories by numerically solving Eq. 9. Trajectories that eventually evolve to ($x^* = 0$), ($x^* = 1$, $r_c^* = \alpha$) or circulate along an interior closed orbit are distinguished by blue, pink and orange, respectively. The emergence of cyclic evolution of group cooperation and local environment (orange areas), which cannot be observed in static global environments, indicates that the global environmental fluctuations could fundamentally alter the evolutionary outcomes in local feedback-evolving game. In all panels, $N = 4$, $\alpha = 1.5$, $\beta = 3.5$, $\theta = 0.5$, $r_d = 0.6$.

Page12(Fig 5)

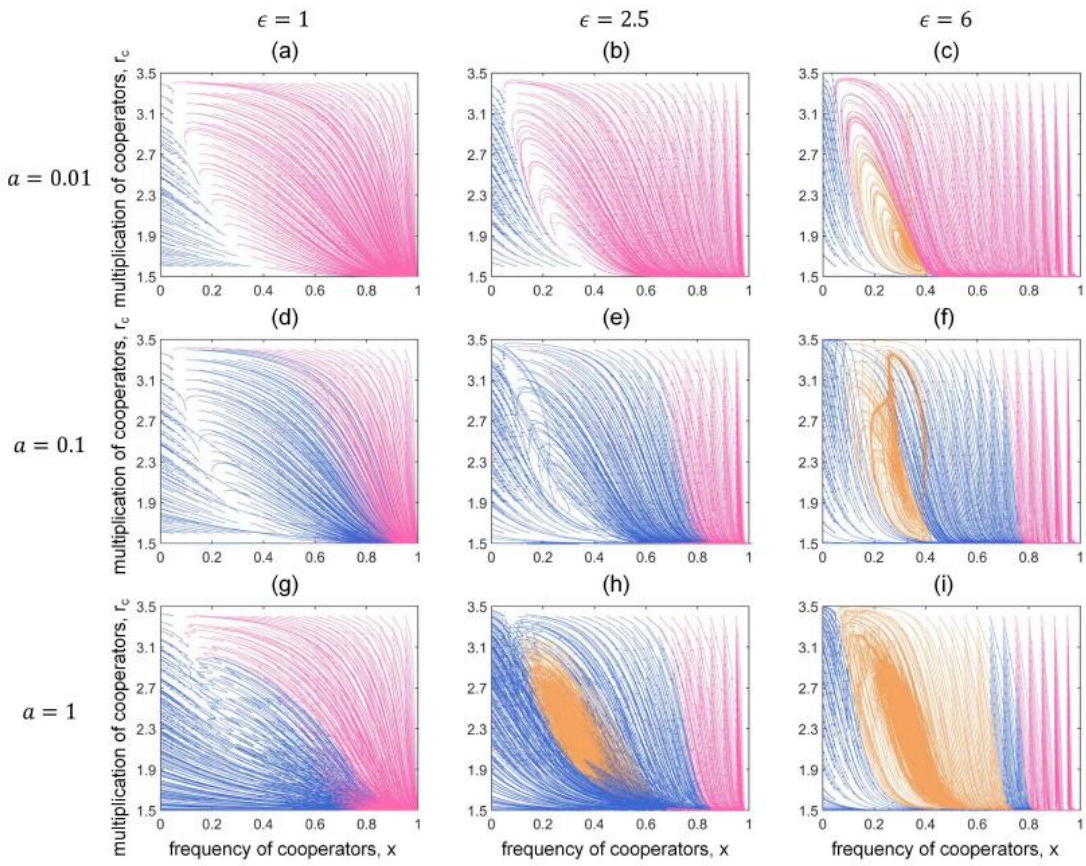

Fig 5. Local game-environment evolution in a continuously changing global environment. We use a periodic continuous function, $w_2(t)$, to describe the global environmental fluctuations. Trajectories that eventually evolve to (x^*= 0), (x^*= 1, r_c^* = α) or circulate along an interior closed orbit are distinguished by blue, pink and orange, respectively. Similarly, the cyclic evolution of group cooperation and local environment (orange areas) arises with larger local feedback speeds ($\epsilon = 6$) and faster global environmental fluctuations (a=1). For easy to compare, the values of parameters in all panels are the same as in Fig 4: N = 4, α = 1.5, β = 3.5, θ = 0.5, r_d = 0.6 and $\delta = -\frac{\pi}{2}$.

Why w_1 is changing between 1.2 and 0.8 and w_2 between 0.5 and 1.5:

Page10(Results)

We also let the expectations of synergistically enhanced or discounted influence exerted by $w_1(t)$ and $w_2(t)$ in time interval T be equal. As a result, we set $w_1(t)$ change between $\frac{1}{T}\int_0^{\mathrm{T}}(1 - 0.5\sin at)\mathrm{d}t \approx 0.7$ and $\frac{1}{T}\int_{\mathrm{T}}^{2\mathrm{T}}(1 - 0.5\sin at)\mathrm{d}t \approx 1.3$.

Page13(Fig 6(a-d) are replotted using the newly updated $w_1(t)$)

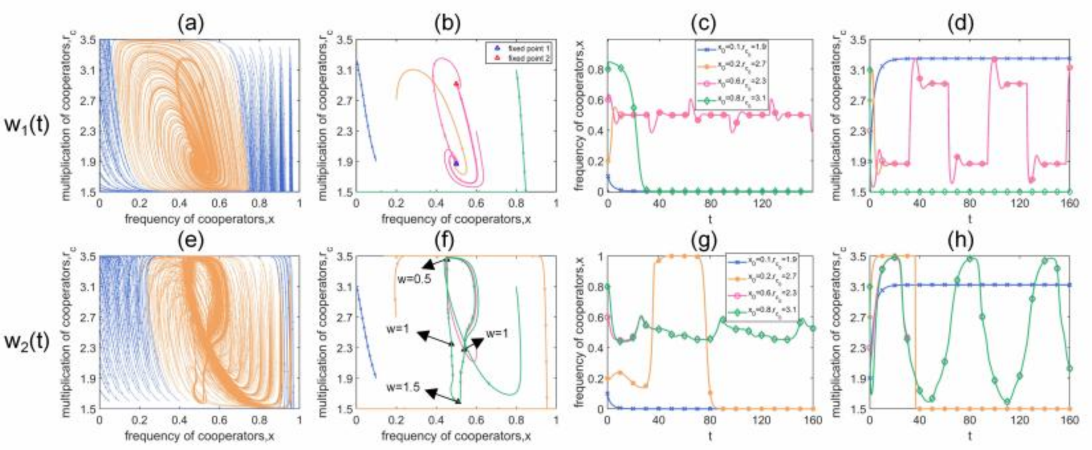

Fig 6. Emergence of cyclic evolution of group cooperation and local environment under periodically changing global environments given by $w_1(t)$ and $w_2(t)$. The first column shows local game-environment evolution similar to Fig 4 and Fig 5. The second column displays four typical dynamic trajectories in detail, particularly the interior closed yet irregular orbits. The last two columns present time evolution of the frequency of cooperators x and the multiplication factor of cooperators r_c under different initial conditions, corresponding to the colored trajectories in the second column. Results show that the formation of cyclic evolution under discrete global environment is due to the fact that the two fixed points are in the mutual attraction domains (b-d), while in continuous situation such phenomena could be interpreted using limit thought (f-h). Parameters: $N = 4$, $\alpha = 1.5$, $\beta = 3.5$, $\theta = 1$, $r_d = 1.2$, $a = 0.1$, $\epsilon = 4$, $\delta = 0$.

2. The authors are trying to make connections to human societal issues. While I appreciate these comparisons, I believe that more biological connections are needed for the readers of this journal. Examples of parts of the paper where I would look for a better-fitting biological illustration are: lines 36-41, extra example in line 45, lines 63-68, 126-129, 143-144, 285-286, 337-338, 340-343. One possibility is to look for these examples in the microbial/systems biology literature. For example, cancer cells or complex bacterial communities.

We appreciate this important question as well as the helpful suggestions, which helps us establish clearer connections between our model and biological systems. We have added illustrations in microbial/systems biology in Introduction, Methods, Results and Conclusion parts. In particular, we provide an explicit example of cancer evolution in Conclusion part to demonstrate the theoretical and practical importance of our work. The detailed revisions in the manuscript are listed as following (The underlined parts are the newly added or revised contents).

New References:

37. Li Y, Wang ZB, Zhang XY, Dang YR, Sun LL, Zhang WP, et al. Experimental evidence for long-term coexistence of copiotrophic and oligotrophic bacteria in pelagic surface seawater. Environmental Microbiology. 2021;23(2):1162–1173.

38. Archetti M, Pienta KJ. Cooperation among cancer cells: applying game theory to cancer.

Nature Reviews Cancer. 2019;19(2):110–117.

48. W¨olfl B, Te Rietmole H, Salvioli M, Kaznatcheev A, Thuijsman F, Brown JS, et al. The contribution of evolutionary game theory to understanding and treating cancer. Dynamic Games and Applications. 2022;12(2):313–342.

62. Sanchez A, Gore J. Feedback between population and evolutionary dynamics determines the fate of social microbial populations. PLoS biology. 2013;11(4):e1001547.

64. Babajanyan S, Koonin EV, Cheong KH. Can Environmental Manipulation Help Suppress Cancer? Non-Linear Competition Among Tumor Cells in Periodically Changing Conditions. Advanced Science. 2020;7(16):2000340.

65. Celiker H, Gore J. Competition between species can stabilize public-goods cooperation within a species. Molecular systems biology. 2012;8(1):621.

66. H Koschwanez J, R Foster K, W Murray A. Sucrose utilization in budding yeast as a model for the origin of undifferentiated multicellularity. PLoS biology. 2011;9(8):e1001122.

70. Glaubitz A, Fu F. Oscillatory dynamics in the dilemma of social distancing. Proceedings of the Royal Society A. 2020;476(2243):20200686.

lines 36-41:

Page2(Introduction)

In microbial systems, cooperation often arises due to the secretion or the release of extracellular enzymes, extracellular antibiotic compounds and growth factors, which modifies the local environmental state that will, in turn, alter the incentive for public goods production [2,4,37,38]. Likewise, in modern society, decision-making dynamics of competitive cognitions can reshape the public opinion environment, especially with the rise of large-scale social networks, and this shared media atmosphere in turn affects the benefit of social discussion in decision-making process [39].

line 45:

Page3(Introduction)

Similar coupled dynamics can also be obtained in psychological-economic systems, social-ecological systems and human-medicine systems [36,38,42,43], relating to a number of big challenges such as global climate change, overfishing, anti-vaccine problems, pandemic prevention and control, cancer treatment [44-48].

lines 63-68:

Page3(Introduction)

In microbial systems, the feedback loop between bacterial evolution and local environment could also arise due to the existence of asymmetric preferential access of public goods, while the changes in culture environment, serving as the exogenous global environment, could remarkably affect their strategic behavior [37,62]

lines 126-129:

Page6(Methods)

For instance, the companies tend to increase the salary and recruit more employees in bull markets, adopting aggressive expansion strategies as each additional staff could provide much more returns, while in contrary, they are more likely to cut salaries and reduce stuff in bear markets. Similarly, changes in nutrient concentration of culture medium or the usage of drugs directly affects the fitness of the competing bacteria or cancer cells [37,38,62,64].

lines 143-144:

Page6(Methods)

To mimic the fact that in many microbial systems, the preferential access mechanism affects the welfare distribution of cooperators and defectors, and that cooperation is first favored and then constrained due to the limitation of total resource and the zero-sum feature of resource consumption, we assume $f(x, r_c)$ is a linear function of cooperators' and defectors' payoffs following the idea in [56]:

$$f(x, r_c) = - x f_c + \theta(1 - x) f_d ,$$

where $x f_c$ and $(1 - x) f_d$ are the expected payoffs for cooperators and defectors, respectively.

lines 285-286:

Page12-13(Results)

Such periodic evolution is actually in line with the intuitions from real world in a way that the group cooperation will neither disappear completely nor always be maintained at the highest level in many complex systems, for instance, the seasonal oscillating dynamics of the COVID-19 infection [67-70], the dynamic adjustment of big companies in the economic cycles and the oscillating abundance of bacteria or cancer cells in periodically varying environments [37,64].

lines 337-338 and lines 340-343:

Page14-15(Discussion)

To sum up, our model provides profound insight into how diverse group behaviors, especially oscillating convergence and cyclic evolution, can emerge from the nonlinear interactions between strategies and dynamic environments. Our work helps understand the complexity of social dilemmas in an ever-changing world. For instance, the game dynamics of the Labor and Capital in social-economic system can be easily translated into our frame: The companies engaging staff can be mapped into the local environment, the state of which coevolves with individual strategies [73]. Outside, the trends of the corresponding industry or the economic state of the country, which can largely determine the marginal benefits of strategic behaviors, can be modeled as the global environment. Such an approach allows us to examine how the evolutionary stable state of the labor-capital game is jointly influenced by factors such as the incentive mechanisms of the company and the development of the economies. Our results also have important practical value in systems biology. Studies have shown that the global periodically varying environment associated with biotic (blood circulation, nutrients supply) or abiotic (periodical drug usage) variations could

induce nonlinear competition between different types of tumor cells in local environments, which drastically changes the evolutionary outcomes and results in oscillating dynamics of tumor population, breaking the dominance of a certain type of tumor cell [64]. Our model incorporating nonlinear local interactions as well as general mechanisms of global environmental changes thus paves ways for a more confined understanding on such dynamical processes of cancer evolution. For instance, the parameters in global environmental changes could be exploited for designing milder control strategies on tumor growth. Similarly, in microbial system, the concentration of casein, corresponding to the global environment, could lead to an oscillation coexistence of copiotroph (W04) and oligotroph (Y09) in the local environment [37]. In microbial experiments, the dilution factor in the growth medium significantly influences the local feedback loop between laboratory yeast population and the dynamics of cooperators, the SUC2 gene [62]. Our model can be used to describe these complex microbial systems and may help in establishing effective control methods that could tune the system evolution to a desired direction.

3. The authors consider two functional forms of w(t), which are in principle very similar (and induce similar behavior). While I understand that the model is already quite complex, I wonder how hard would it be to analyze an example when w(t) also depends on x? Could it be that the global environmental conditions are also dependent on the frequency of cooperators, for example, in a threshold-function manner?

We thank the reviewer for this constructive suggestion, which extends the generality of our model. We have conducted additional analysis under a new threshold-function $\mathrm{w}_4(\mathrm{t})$ which is also frequency-dependent in S3 Appendix. We find that unlike the time-dependent cases, the internal periodic orbit disappears, instead, an interior stable equilibrium emerges under different threshold parameters. We have also added a brief additional introduction at the end of the Results part. The manuscript has been updated as follows.

Page13(Results)

Note that in this paper the global environment is assumed to be time-dependent. In real-world complex systems, the global environment may exhibit more complicated manners. We provide a simple additional analysis where the global environment is also frequency-dependent and is in a threshold-function manner in S3 Appendix.

Supporting Information(S3 Appendix)

Next, we examine how the evolutionary outcomes would be influenced when the global environment exhibits more complicated manners. We study a simple case where the global environment is also frequency-dependent and is in a threshold-function manner, which is denoted by $w_4(t)$:

$$w_4(t) = \begin{cases} 1.3, & x(t) < K \\ 0.7, & x(t) \geq K \end{cases}$$

where K is a threshold value between 0 and 1. When the frequency of cooperators $x(t)$ is smaller than the threshold K, the global environment becomes synergistically enhanced in order to facilitate cooperation, while on the contrary, when $x(t)$ is larger than K, the lobal environment becomes discounted. Figure S7 shows local game-environment evolution under these

frequency-dependent global environments. Surprisingly, we find that unlike the time-dependent cases, the internal periodic orbit disappears, instead, an interior stable equilibrium (where the grey trajectories stabilize in Figure S7(a)-(c)) emerges under different thresholds. We further present the detailed evolutionary trajectories of several representative initial points in Figure S7(d)-(f). The phase plane is divided into two regions by the threshold K, with orange on the left representing $w(t)$ = 1.3 and blue on the right representing $w(t)$ = 0.7. The triangles represent the stable interior fixed points, and the vertical dotted lines indicate $x=\frac{\theta}{\theta+1}$, which is also the abscissa of the interior fixed points. Obviously $x=\frac{\theta}{\theta+1}$ can only fail into one of the two regions, either orange or blue, and the corresponding $w(t)$ decides the final position of the interior stable equilibrium. In other words, the system can only have one interior fixed point in our threshold model. It is also worthy of note that a stable boundary equilibrium may emerge when the threshold K is large (Figure S7(c) and (f)).

Supporting Information(Figure S7)

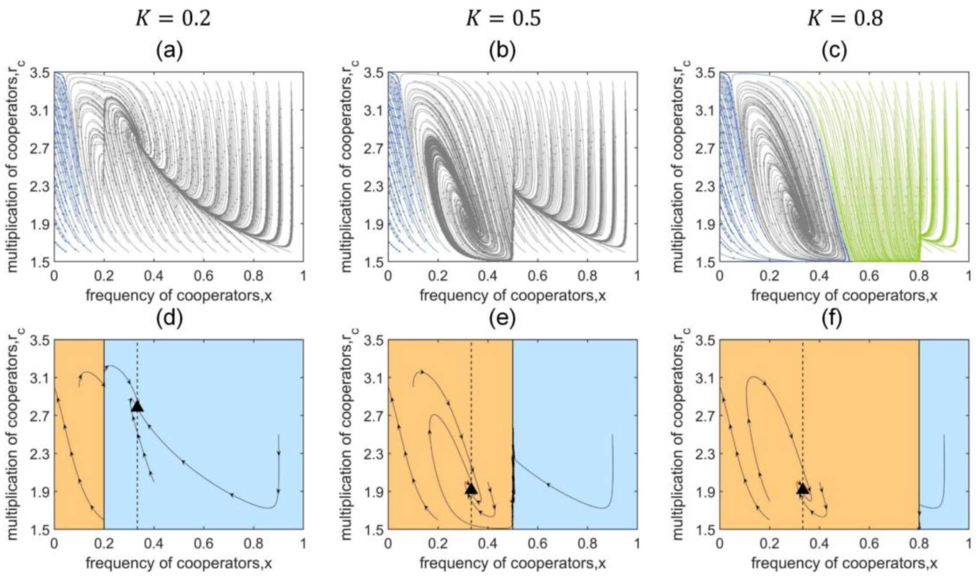

Figure S7. Local game-environment evolution under frequency-dependent global environments. We assume the global environment is in a simple threshold-function manner denoted by $w_4(t)$. In specific, when the frequency of cooperators $x(t)$ is smaller than the threshold K, the global environment becomes synergistically enhanced in order to facilitate cooperation, while on the contrary, when $x(t)$ is larger than K, the global environment becomes discounted. The first row shows the overall evolutionary trends. Trajectories that eventually evolve to full defection, the stable interior fixed point and the boundary equilibrium point on the x-axis are distinguished by blue, grey and green, respectively. The second row shows the detailed evolutionary trajectories of several representative initial points in the phase plane. The plane is divided into two regions by the threshold K, with orange on the left representing $w(t)$ = 1.3 and blue on the right representing $w(t)$ = 0.7. The vertical dotted and solid lines represent $x=\frac{\theta}{\theta+1}$ and x = K, respectively, and the triangle represents the stable interior fixed point. Unlike the time-dependent cases, the internal periodic orbit disappears, instead, an interior stable equilibrium emerges under all thresholds. For

comparison, the parameters are the same as in Fig 4: $N = 4$, $\alpha = 1.5$, $\beta = 3.5$, $\theta = 0.5$, $r_d = 0.6$, $\epsilon = 6$.

4. As a general comment about figures, I found the text in figures too small making it hard to read. The authors might save some space by placing the panels closer to each other and naming axis only once per row/column in the figure. In addition, while I do not have strong feelings about it, it might be worth separating axis in fig. 2(b) as frequency x runs only up to 1. It might also be worth it to increase the thickness of lines whenever possible.

We thank the reviewer for these suggestions on our figures. We have modified the figures in the manuscript correspondingly. First, we increase the font size of the text in Fig 2 and Fig 6. Second, we name axis only once per row/column in Fig 3-5 to place the panels closer. Third, while we have tried to separate axis in Fig 2(b), we find the multiple intersection points looks a bit messy. We therefore choose to add a new line x=1 in dashed black to show the superior limit of x. Last, we increase the thickness of lines in all figures.

5. Generally, I think that the paper needs some proof-reading. I provide a list of typos/corrections, but please note that it is not exhaustive:

- Line 14: the heterogeneity of players

Thank you, we have modified this word.

- Line 19: Accordingly might be not the right word here

Thank you, we have replaced "Accordingly" with "Therefore".

- Line 33: a wide range

Thank you, we have corrected this phrase.

- Line 35: "and etc" seems a bit too casual

We thank the reviewer for this suggestion. We have deleted "and etc" and added new content to refine this background.

Page2(Introduction)

In microbial systems, cooperation often arises due to the secretion or the release of extracellular enzymes, extracellular antibiotic compounds and growth factors, which influence the local environmental state that will, in turn, alter the incentive for public goods production.

- Line 43: socio-ecological (this comment also applies to all other instances of sociosomething)

Thank you, we have corrected all these words.

- Line 179: in a skilled project,

Thank you, we have added the word "in".

- Line 258: evolution (singular, similar to other instances in the manuscript)

Thank you, we have corrected all these words in the manuscript.

- Line 297: on the other hand

Thank you, we have corrected this phrase.

- Line 305: furthermore might be not the best word here

Thank you, we have replaced “Furthermore” with “Meanwhile”.

- Line 314: the cooperators

Thank you, we have corrected it.

- Line 321: global dynamic environments?

Thank you, we have adjusted the order of these two words.

- To beautify the numbers a bit, can the authors try to find rational numbers representing the special values of parameters? Say, 0.4444 can be written as 4/9

Thank you, we have beautified the values of the parameters. Owing to the complex nonlinearity in Eq. 12, it is hard to get rational numbers in all circumstances. Therefore, we choose to retain two decimal places for all the special numbers.

Reviewer #3:

The authors develop a model of an eco-evolutionary game where there is both endogenous environmental feedback and exogenous environmental change. This combination of factors would be applied to strategic settings where there is seasonality or where the game is embedded within a larger system that exerts forcing on the local game-environment dynamics. By combining these exogenous and endogenous drivers of environmental change, the paper makes a nice contribution to evolutionary game theory.

The game that they study is an environmentally dependent public goods game with two relevant 'environmental' factors. First is the multiplicative factor of the public good that cooperators experience, which depends on endogenous feedback from the strategies that people use. Second is the curvature of the payoffs as a function of the number of cooperators which can be either concave or convex depending on the state of an exogenously changing global environment. As the global environment fluctuates, there are alternating periods with increasing and decreasing marginal returns to cooperation.

My overarching comment in the manuscript concerns the connections that the authors make between their model and its practical implications. Lines 333-343 state that this model shows how complex dynamics can emerge from nonlinear interactions among strategies and the environment. I fully agree with the authors on this claim. However, they also assert that the model has 'practical value for solving social dilemmas is an ever-changing world'. In the introduction, many systems which exhibit social dilemmas are discussed, however this does not carry through to the remainder of the manuscript. While the authors do at times relate their model to such systems (e.g. crowdsourcing; skilled vs unskilled collaboration; corporate hiring practices), none of these examples are dealt with in sufficient depth to demonstrate how this model could be used to solve social dilemmas in practice. I challenge the authors to develop this aspect of their work, so the manuscript can more clearly demonstrate its theoretical and practical importance.

We thank the reviewer for pointing out this important question. We totally agree with the criticism that how our model can be used to solve social dilemmas in practice is unclear. Firstly, we have made substantial revisions in the Conclusion part to reclaim the practical implications of our model. In particular, we give two detailed examples, one demonstrates how our model can be used to study the game dynamics of the Labor and Capital in social-economic system, the other shows potential applications of our framework in the study of cancer evolution. Note that we have deleted the claim of "solving social dilemmas", instead, we stress the potentiality of our model in designing effective control strategies in microbial systems. Second, some examples that illustrate the connections of our model and systems biology are also added into Introduction, Methods, Results and Conclusion parts. The detailed modifications are as follows. (The underlined parts are the newly added or revised contents).

New References:

37. Li Y, Wang ZB, Zhang XY, Dang YR, Sun LL, Zhang WP, et al. Experimental evidence for long-term coexistence of copiotrophic and oligotrophic bacteria in pelagic surface seawater. Environmental Microbiology. 2021;23(2):1162–1173.

38. Archetti M, Pienta KJ. Cooperation among cancer cells: applying game theory to cancer.

Nature Reviews Cancer. 2019;19(2):110–117.

48. W¨olfl B, Te Rietmole H, Salvioli M, Kaznatcheev A, Thuijsman F, Brown JS, et al. The contribution of evolutionary game theory to understanding and treating cancer. Dynamic Games and Applications. 2022;12(2):313–342.

62. Sanchez A, Gore J. Feedback between population and evolutionary dynamics determines the fate of social microbial populations. PLoS biology. 2013;11(4):e1001547.

64. Babajanyan S, Koonin EV, Cheong KH. Can Environmental Manipulation Help Suppress Cancer? Non-Linear Competition Among Tumor Cells in Periodically Changing Conditions. Advanced Science. 2020;7(16):2000340.

70. Glaubitz A, Fu F. Oscillatory dynamics in the dilemma of social distancing. Proceedings of the Royal Society A. 2020;476(2243):20200686.

Page2(Introduction)

In microbial systems, cooperation often arises due to the secretion or the release of extracellular enzymes, extracellular antibiotic compounds and growth factors, which modifies the local environmental state that will, in turn, alter the incentive for public goods production [2,4,37,38]. Likewise, in modern society, decision-making dynamics of competitive cognitions can reshape the public opinion environment, especially with the rise of large-scale social networks, and this shared media atmosphere in turn affects the benefit of social discussion in decision-making process [39].

Page3(Introduction)

In microbial systems, the feedback loop between bacterial evolution and local environment could also arise due to the existence of asymmetric preferential access of public goods, while the changes in culture environment, serving as the exogenous global environment, could remarkably affect their strategic behavior [37,62]

Page6(Methods)

For instance, the companies tend to increase the salary and recruit more employees in bull markets, adopting aggressive expansion strategies as each additional staff could provide much more returns, while in contrary, they are more likely to cut salaries and reduce stuff in bear markets. Similarly, changes in nutrient concentration of culture medium or the usage of drugs directly affects the fitness of the competing bacteria or cancer cells [37,38,62,64].

Page12-13(Results)

Such periodic evolution is actually in line with the intuitions from real world in a way that the group cooperation will neither disappear completely nor always be maintained at the highest level in many complex systems, for instance, the seasonal oscillating dynamics of the COVID-19 infection [67-70], the dynamic adjustment of big companies in the economic cycles and the oscillating abundance of bacteria or cancer cells in periodically varying environments [37,64].

Page14-15(Discussion)

To sum up, our model provides profound insight into how diverse group behaviors, especially

oscillating convergence and cyclic evolution, can emerge from the nonlinear interactions between strategies and dynamic environments. Our work helps understand the complexity of social dilemmas in an ever-changing world. For instance, the game dynamics of the Labor and Capital in social-economic system can be easily translated into our frame: The companies engaging staff can be mapped into the local environment, the state of which coevolves with individual strategies [73]. Outside, the trends of the corresponding industry or the economic state of the country, which can largely determine the marginal benefits of strategic behaviors, can be modeled as the global environment. Such an approach allows us to examine how the evolutionary stable state of the labor-capital game is jointly influenced by factors such as the incentive mechanisms of the company and the development of the economies. Our results also have important practical value in systems biology. Studies have shown that the global periodically varying environment associated with biotic (blood circulation, nutrients supply) or abiotic (periodical drug usage) variations could induce nonlinear competition between different types of tumor cells in local environments, which drastically changes the evolutionary outcomes and results in oscillating dynamics of tumor population, breaking the dominance of a certain type of tumor cell [64]. Our model incorporating nonlinear local interactions as well as general mechanisms of global environmental changes thus paves ways for a more confined understanding on such dynamical processes of cancer evolution. For instance, the parameters in global environmental changes could be exploited for designing milder control strategies on tumor growth. Similarly, in microbial system, the concentration of casein, corresponding to the global environment, could lead to an oscillation coexistence of copiotroph (W04) and oligotroph (Y09) in the local environment [37]. In microbial experiments, the dilution factor in the growth medium significantly influences the local feedback loop between laboratory yeast population and the dynamics of cooperators, the SUC2 gene [62]. Our model can be used to describe these complex microbial systems and may help in establishing effective control methods that could tune the system evolution to a desired direction.

Comments:

-When the model setup is introduced, it is stated that there is an infinite population that is well mixed, but also that the game has N participants. If I understand correctly, the PGG is played in random groups of N that are drawn from an infinite population. However, I think readers would benefit from the manuscript being more explicit about the assumptions that lead to the payoffs and culminate in equation 6.

We thank the reviewer for this suggestion. We have added more explicit description of the modeling assumptions. The manuscript has been modified as follows.

Page4-5(Methods)

In every game, each of the N participants randomly drawn from the infinite population can choose to be a cooperator contributing c to the public pool, or a defector reaping without sowing.

-When synergy vs discounting is introduced, it may be more intuitive for many readers to explain how different values of w impact the concavity of P_d(k) and P_c(k). The authors describe these functions as being either ‘synergistic’ or ‘discounting’. A minor comment is that the latter of these terms is often used to describe intertemporal aggregation of payoffs in economics and so it’s use in this paper may cause confusion among some readers.

Thank you, we have modified some descriptions in this part based on your suggestions. The parameter w was first introduced in a previous paper [61]. In their work, they use the words "synergy" and "discounting" to represent accumulated benefits of different w. Here, in order to avoid confusion from the potential readers, we reserve their statement, cite their work and introduce two examples to explain "synergistically enhanced" and "discounted". We also briefly conclude how different w impact the concavity of $P_d(k)$ and $P_c(k)$. The manuscript has been updated as follows.

Reference:

61. Hauert C, Michor F, Nowak MA, Doebeli M. Synergy and discounting of cooperation in social dilemmas. Journal of theoretical biology. 2006;239(2):195–202.

Page5(Methods)

such that the benefits created by each additional cooperator are rescaled, either synergistically enhanced when $w > 1$, or discounted when $w < 1$. For instance, as the concentration of enzymes produced by cooperators increases, the enzyme-mediated reaction exhibits a faster rate than linearity, indicating that additional enzyme production has enhanced payoffs ($w > 1$). On the contrary, the benefit provided by the first cooperator in foraging yeast cells may be critical for survival, whereas the value of additional food decreases ($w < 1$) [61]. Therefore, $P_d(k)$ and $P_c(k)$ are convex when $w > 1$ and are concave when $w < 1$. Finally, when $w = 1$, the classic linear PGG can be recovered.

-Equation 8 defines f(x,r_c), which drives the dynamics of r_c. However, I found the presentation of this equation to be confusing and the text did not help me generate intuition about why this function is used. It would be really helpful if the authors could provide a more intuitive explanation for the why f(x,r_c) takes the form it does, and what it means.

We thank the reviewer for this suggestion. We have tried to add a more intuitive explanation for the form of $f(x, r_c)$, which reads as follows.

Page6(Methods)

To mimic the fact that in many microbial systems, the preferential access mechanism affects the welfare distribution of cooperators and defectors, and that cooperation is first favored and then constrained due to the limitation of total resource and the zero-sum feature of resource consumption, we assume $f(x, r_c)$ is a linear function of cooperator's and defector's payoffs following the idea in [56]:

$$f(x, r_c) = -xf_c + \theta(1-x)f_d\,,$$

where xf_c and $(1-x)f_d$ are the expected payoffs for cooperators and defectors, respectively. $\theta > 0$ denotes the distribution ratio of the expected total payoff of the cooperators and defectors. Such local feedback-evolving dynamics could facilitate cooperation by increasing r_c when x is small, while in turn, a relatively large x leads to the decrease of cooperator's rewards, subjecting to the law of diminishing marginal utility.

-Figure 2 presents results for the model with a static values of w. It would be great if the figure caption contained enough detail so that a reader could interpret the results shown in the figure

without having to refer to the text of the MS. Right now, the caption doesn't explain that the threshold epsilon* is the minimum value that leads to internal stability, or detail why the blue line in panel b becomes dashed and what this means.

We thank the reviewer for this helpful comment. We have replotted Fig 2 and modified the caption according to your suggestions. The new caption contains more detailed information of the figure, including the meaning of threshold ϵ^* and the interpretation of the dashed blue line. The manuscript has been updated as follows.

Page7(Fig 2)

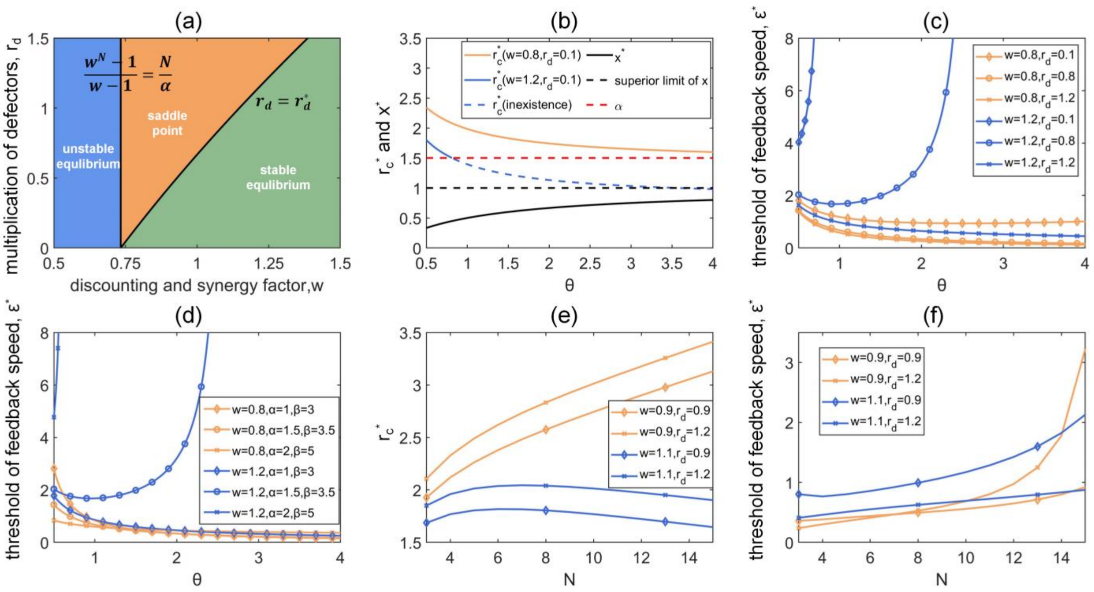

Fig 2. Effects of varying modeling parameters on equilibrium states of local game-environment evolution in static global environments. Panel (a) shows the combined influence of w and r_d on the stability of the boundary fixed point $(1, \alpha)$. Panel (b) presents the trends of the interior fixed point (x^*, r_c^*) as θ changes (the dashed blue line indicates that the value of r_c^* is out of parameter range $[\alpha, \beta]$). Panel (c) and (d) show how the threshold of the relative feedback speed ϵ^*, which is the minimum value leading to the interior stability, is affected by different parameters. Panel (e) and (f) show the detailed influence of group size N on the equilibrium states revealed by r_c^* and ϵ^*, respectively. Parameters: $N = 4$ in (a-d), $\alpha = 1.5, \beta = 3.5$ in (a-c, e-f) and $r_d = 0.8$ in (d).
