# Supplementary material for: Nonlinear eco-evolutionary games with global environmental fluctuations and local environmental feedbacks
Source: PLoS Comput Biol. 2023 Jun 28;19(6):e1011269. doi: 10.1371/journal.pcbi.1011269 (PMC10335700; doi:10.1371/journal.pcbi.1011269)
Supplement: Attachment — Submitted filename: report_revision.pdf [file pcbi.1011269.s006.pdf]

First of all, I would like to thank the authors for all the effort they put into the revision of the paper. I believe that the new results they obtained are quite important and demonstrate well how the model works. As a result, I am not quite sure these new results (especially, the results on the frequency-dependent functional form of w(t)) should be reported in the SI. I would very strongly advise putting them in the main text. The reason for this is that I believe that functional forms w_1(t) and w_2(t) are very similar and exhibit similar behavior of the dynamics. Hence, I would instead report results for w_1(t) and w_4(t) in the main text and put w_2(t) in the SI as a robustness check with respect to the continuity of the functional form. However, I leave this decision to the editor.

My more minor comments are as follows:

1. The paper still needs very careful proofreading, especially the new added parts.
2. Fig.2 I would recommend reorganizing as follows: in the first row put panels (a), (e) and (f), in the second row put panels (b), (c), (d) so that readers could compare them better (this would sort the panels by the x axis).
3. I would also like to see figures S2 and S3 in the main text.
