# Supplementary material for: Nonlinear eco-evolutionary games with global environmental fluctuations and local environmental feedbacks
Source: PLoS Comput Biol. 2023 Jun 28;19(6):e1011269. doi: 10.1371/journal.pcbi.1011269 (PMC10335700; doi:10.1371/journal.pcbi.1011269)
Supplement: Attachment — Submitted filename: renamed_43883.pdf [file pcbi.1011269.s007.pdf]

Point-by-point response to the reviewers' comments

Manuscript PCOMPBIOL-D-22-01825R1

Reviewer #1:

The authors have revised their manuscript based on the comments. I would suggest acceptance of the updated version.

We are very pleased that you are satisfied with our modifications in response to your valuable suggestions, which have greatly improved our work. And thanks again for your professional review.

Reviewer #2:

First of all, I would like to thank the authors for all the effort they put into the revision of the paper. I believe that the new results they obtained are quite important and demonstrate well how the model works. As a result, I am not quite sure these new results (especially, the results on the frequency-dependent functional form of w(t)) should be reported in the SI. I would very strongly advise putting them in the main text. The reason for this is that I believe that functional forms w_1(t) and w_2(t) are very similar and exhibit similar behavior of the dynamics. Hence, I would instead report results for w_1(t) and w_4(t) in the main text and put w_2(t) in the SI as a robustness check with respect to the continuity of the functional form. However, I leave this decision to the editor.

We thank the reviewer for the positive feedback and this constructive suggestion. We have put the new results on frequency-dependent functional form of w(t) in the main text. In addition, w_2(t) has been reported in the SI as a robustness check. The corresponding parts are updated as follows.

Page1(Abstract)

The intricate interplay between strategic behavior and environment is ubiquitous in complex systems of different scales. Previous works mainly focus on one aspect of the environmental changes: either global environment fluctuations that unidirectionally decide the welfare of the evolutionary dynamics, or local environment feedbacks that coevolve with the strategic behavior. Here we develop a theoretical framework that integrates them both in order to obtain a more complete picture of how group cooperation evolves in a general dynamic environment. We show that global environmental fluctuations can fundamentally alter the dynamical predictions of local game-environment evolution. The most interesting finding is the emergence of cyclic evolution of group cooperation and local environment, which forms an interior irregular loop in the phase plane, depending on the relative changing speed of both global and local environments compared to the strategic change. <u>Such irregular loop, however, is substituted by an interior stable fixed point when considering a more complicated situation where the global environment is also frequency-dependent.</u> Our results show how rich dynamical outcomes arise from the interactions between strategic behaviors and their natural or social environments, which has important practical value for solving social dilemmas in an ever-changing world.

Page4(Introduction)

Surprisingly, such eco-evolutionary dynamics could form an interior closed yet irregular orbit in the phase plane, depending on the relative time-scale of both global and local environments versus strategic changes. <u>However, when the global environment is not only time-dependent but also frequency-dependent, such interior closed orbit is substituted by an interior stable fixed point</u>. Our results provide novel insights toward how complex group behaviors emerge from the nonlinear interactions between strategies and dynamic environments, especially in a non-autonomous system, which is important for understanding the evolution of social dilemmas in a changing world.

Page14(Fig 7)

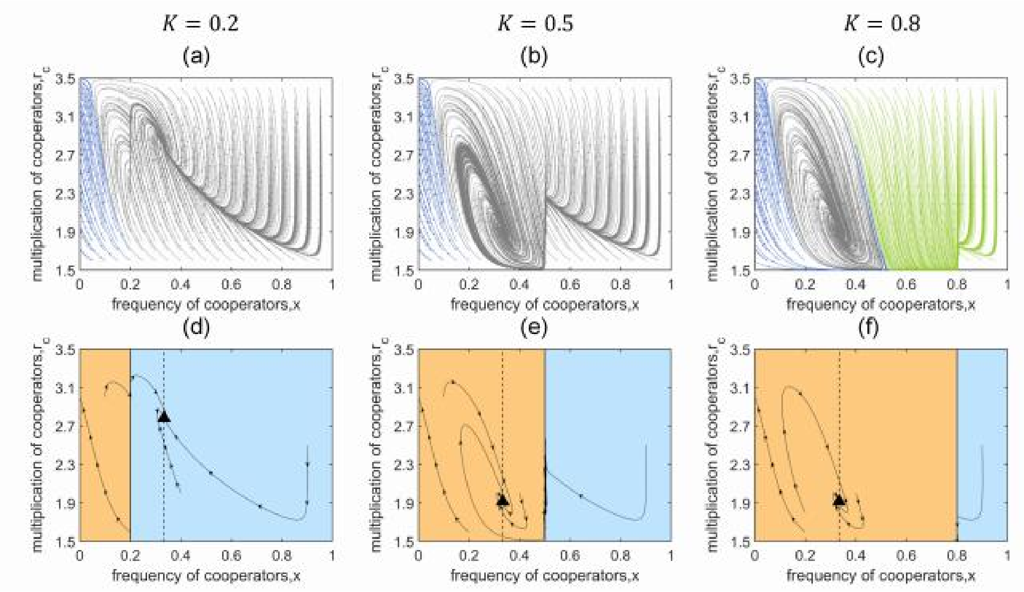

Fig 7. Local game-environment evolution under frequency-dependent global environments. We assume the global environment is in a simple threshold-function manner denoted by $w_4(t)$. In specific, when the frequency of cooperators $x(t)$ is smaller than the threshold K, the global environment becomes synergistically enhanced in order to facilitate cooperation, while on the contrary, when $x(t)$ is larger than K, the global environment becomes discounted. The first row shows the overall evolutionary trends. Trajectories that eventually evolve to full defection, the stable interior fixed point and the boundary equilibrium point on the x-axis are distinguished by blue, grey and green, respectively. The second row shows the detailed evolutionary trajectories of several representative initial points in the phase plane. The plane is divided into two regions by the threshold K, with orange on the left representing $w(t)$ = 1.3 and blue on the right representing $w(t)$ = 0.7. The vertical dotted and solid lines represent $x = \frac{\theta}{\theta+1}$ and $x = K$, respectively, and the triangle represents the stable interior fixed point. Unlike the time-dependent cases, the internal periodic orbit disappears, instead, an interior stable equilibrium emerges under all thresholds. For comparison, the parameters are the same as in Fig 4: $N = 4$, $\alpha = 1.5$, $\beta = 3.5$, $\theta = 0.5$, $r_d = 0.6$, $\epsilon = 6$.

Page13-14(Results)

For now, the global environment is only assumed to be time-dependent. In real-world complex systems, the global environment could certainly exhibit more complicated manners. As a heuristic case study, we further examine how the evolutionary outcomes are influenced when the global environment is also frequency-dependent and is in a threshold-function manner, which is denoted by $w_2(t)$:

$$w_2(t) = \begin{cases} 1.3, & x(t) < K \\ 0.7, & x(t) \geq K \end{cases}$$

where K is a threshold value between 0 and 1. When the frequency of cooperators $x(t)$ is smaller

than the threshold K, the global environment becomes synergistically enhanced in order to facilitate cooperation, while on the contrary, when $x(t)$ is larger than K, the global environment becomes discounted. Figure S7 shows local game-environment evolution under these frequency-dependent global environments. Surprisingly, we find that unlike the time-dependent cases, the internal periodic orbit disappears, instead, an interior stable equilibrium (where the grey trajectories stabilize in Fig 7(a)-(c)) emerges under different thresholds. We further present the detailed evolutionary trajectories of several representative initial points in Fig 7(d)-(f). The phase plane is divided into two regions by the threshold K, with orange on the left representing w(t) = 1.3 and blue on the right representing w(t) = 0.7. The triangles represent the stable interior fixed points, and the vertical dotted lines indicate $x = \frac{\theta}{\theta+1}$, which is also the abscissa of the interior fixed points. Obviously $x = \frac{\theta}{\theta+1}$ can only fail into one of the two regions, either orange or blue, and the corresponding $w(t)$ decides the final position of the interior stable equilibrium. In other words, the system can only have one interior fixed point in our threshold model. It is also worthy of note that a stable boundary equilibrium may emerge when the threshold K is large (Fig 7(c) and (f)).

Page15(Discussion)

We unveil that this new dynamical phenomenon can be intuitively understood as a limit of the continuously converging process of the dynamical paths confined by the fixed points. Importantly, our theoretical framework has shown the crucial role of relative time-scales of both global and local environments compared to strategic interactions. Further, when examining a more intricate scenario where the global environment is also frequency-dependent, we find an interior stable equilibrium instead of cyclic evolution emerges, underscoring the significance of the nature of the global environment.

Page16(Discussion)

Though in this work we only consider the periodic fluctuations and a simple threshold-function manner of the global environment which are widespread in natural systems, various changing rules can be studied by simply giving different time-evolving functions $w(t)$.

My more minor comments are as follows:

1. The paper still needs very careful proofreading, especially the new added parts.

Thank you. The manuscript has been thoroughly proofread from begging to end. The modifications are highlighted correspondingly.

Abstract

(1) ‘Our results provide important insights toward......’ has been modified as ‘Our results provide important insights into......’.

Introduction

(2) ‘Naturally, understanding why the persistent cooperation occurs ubiquitously, on what conditions a stable cooperation could be maintained or promoted......’ has been modified as

‘Naturally, understanding why persistent cooperation occurs ubiquitously, under what conditions stable cooperation could be maintained or promoted......’.

(3) ‘Therefore, coevolutionary games that incorporate the evolution process of an exogenous environment have been largely studied’ has been modified as ‘Therefore, coevolutionary games incorporating an exogenous environment's evolution process have been largely studied’.

(4) ‘Note that ...... is independent from the strategic interactions’ has been modified as ‘Note that ...... is independent of the strategic interactions’.

(5) ‘In crowdsourcing projects, cooperations can emerge from the asymmetric incentive feedback which results in a local feedback loop’ has been modified as ‘In crowdsourcing projects, cooperation can emerge from the asymmetric incentive feedback, resulting in a local feedback loop’.

(6) ‘due to the existence of asymmetric preferential access of public goods’ has been modified as ‘due to the existence of asymmetric preferential access to public goods’.

Methods

(7) ‘the former is common in biology and the later has been largely revealed in economics’ has been modified as ‘the former is common in biology and the latter has been largely revealed in economics’.

(8) ‘while in contrary’ has been modified as ‘on the contrary’.

Results

(9) ‘Correspondingly, the eco-evolutionary game described by Eq. \ref{eq9} degenerates to an autonomous system’ has been modified as ‘Correspondingly, the eco-evolutionary game described by Eq. \ref{eq9} degenerates into an autonomous system’.

(10) ‘Of particular interest, we analyze on what condition......’ has been modified as ‘Of particular interest, we analyze under what condition......’.

(11) ‘regardless the changes in N’ has been modified as ‘regardless of the changes in N’.

(12) ‘with higher reward for each member’ has been modified as ‘with higher rewards for each member’.

(13) ‘Specifically, we focus on periodic global environmental fluctuations, which is typical and widespread in various complex systems’ has been modified as ‘Specifically, we focus on periodic global environmental fluctuations, which are typical and widespread in various complex systems’.

(14) ‘and etc’ has been modified as ‘, ect’.

(15) ‘ under a discretely varying global environments’ has been modified as ‘ under a discretely varying global environment’.

(16) ‘the local eco-evolutionary dynamics evolves’ has been modified as ‘the local eco-evolutionary dynamics evolve’.

(17) ‘These results just show......, especially highlight......’ has been modified as ‘These results just

show......, especially highlighting......'.

Discussion

(18) '......control strategies on tumor growth' has been modified as '......control strategies for tumor growth'.

2. Fig.2 I would recommend reorganizing as follows: in the first row put panels (a), (e) and (f), in the second row put panels (b), (c), (d) so that readers could compare them better (this would sort the panels by the x axis).

We thank the reviewer for this comment. We have reorganized Fig 2 and the manuscript has been updated as follows.

Page7(Fig 2)

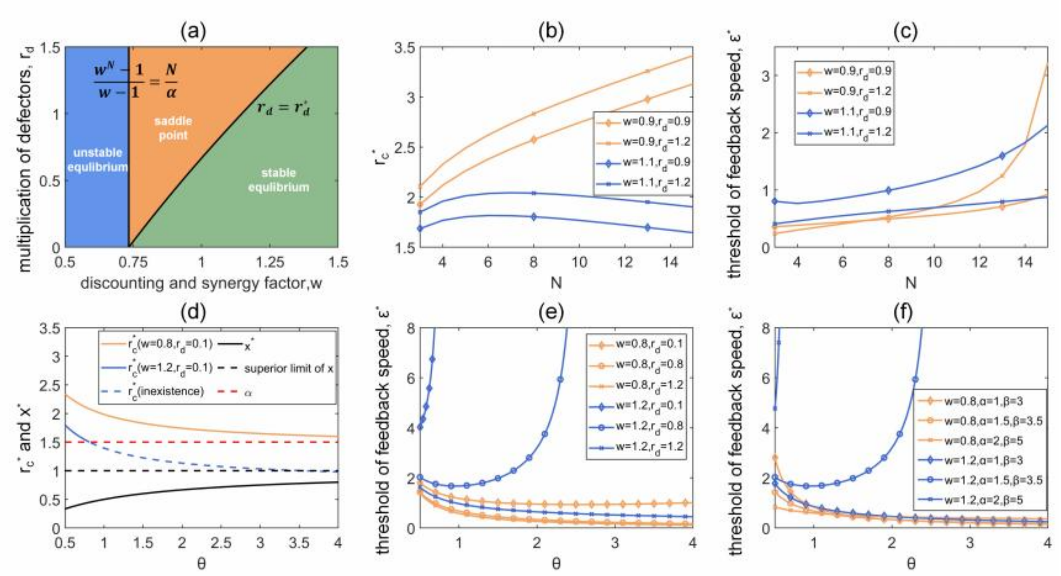

Fig 2. Effects of varying modeling parameters on equilibrium states of local game-environment evolution in static global environments. Panel (a) shows the combined influence of w and r_d on the stability of the boundary fixed point $(1, \alpha)$. Panel (b) and (c) show the detailed influence of group size N on the equilibrium states revealed by r_c^* and ϵ^*, respectively. Panel (d) presents the trends of the interior fixed point (x^*, r_c^*) as θ changes (the dashed blue line indicates that the value of r_c^* is out of parameter range $[\alpha, \beta]$). Panel (e) and (f) show how the threshold of the relative feedback speed ϵ^*, which is the minimum value leading to the interior stability, is affected by different parameters. Parameters: $N = 4$ in (a, d-f), $\alpha = 1.5$, $\beta = 3.5$ in (a, c, e) and $r_d = 0.8$ in (f).

Page8-9(Results)

Of particular interest, we analyze under what condition the interior equilibrium point

$$(x^* = \frac{\theta}{\theta + 1}, r_c^* = \frac{N(w-1) + r_d\left((wx^* - x^* + 1)^{N-1} - 1\right)}{w(wx^* - x^* + 1)^{N-1} - 1}), \tag{10}$$

could be stable, bringing about the coexistence of cooperators and defectors with an intermediate local environmental state. Since α<r_c*<β, we first identify the existence condition of this equilibrium point

$$\max\left\{0,\frac{\alpha\left(w\left(\frac{w\theta+1}{\theta+1}\right)^{N-1}-1\right)-N\left(w-1\right)}{\left(\frac{w\theta+1}{\theta+1}\right)^{N-1}-1}\right\}\leq r_d$$
$$\leq\min\left\{\alpha,\frac{\beta\left(w\left(\frac{w\theta+1}{\theta+1}\right)^{N-1}-1\right)-N\left(w-1\right)}{\left(\frac{w\theta+1}{\theta+1}\right)^{N-1}-1}\right\}. \tag{11}$$

Furthermore, the existing interior fixed point is stable only if the relative feedback speed of the local environment ε exceeds a threshold ε*, which can be written as follows:

$$\epsilon^*=\frac{\left(r_c^*w-r_d\right)\left(N-1\right)\left(w\theta+1\right)^{N-2}\left(w-1\right)}{\left(r_c^*-\alpha\right)\left(\beta-r_c^*\right)\left[w(w\theta+1)^{N-1}-(1+\theta)^{N-1}\right]}. \tag{12}$$

Then, we show how the group size N influences the equilibrium states of local game-environment evolution revealed by r_c* and ε*, respectively (Fig 2(b) and (c)). To ensure that the interior fixed point always exists as N changes, we set w=0.9 and 1.1, r_d=0.9 and 1.2, θ=2. We find that in a discounting global environment, r_c* is approximately in direct proportion to N, while in the synergy condition, r_c* increases first and then decreases gradually as N increases (Fig 2(b)). Interestingly, r_c* is always larger when w is smaller or r_d is larger regardless of the changes in N. This is intuitive: when the global environment is worse or the multiplication factor of defectors is larger, the payoff coefficient of cooperators should be increased in order to maintain the stable coexistence of cooperators and defectors. We also find that ε* increases as N increases under all four sets of parameters, indicating that the emergence of the stable interior fixed point requires faster local environmental feedback when the group size becomes larger (Fig 2(c)).

As shown by Eq. 10, the final frequency of cooperator x* is solely determined by θ, the distribution ratio of cooperator's and defector's total payoffs. As θ increases, x* also increases whereas the stable multiplication factor of cooperators r_c* decreases (Fig 2(d)). Such changing trends are intuitive in many teamworks: an unskilled project, which has a relatively larger θ, often requires more participants with lower benefits. On the contrary,, in a skilled project such as scientific collaboration, the decrease of θ results in small research teams with higher rewards for each member.

Further, Fig 2(e) shows how ε* varies as w, r_d and θ change. In a less optimistic global environment where the group benefits are discounted (w=0.8), ε* gently decreases as θ increases. In a booming global environment where the group benefits are synergistically enhanced (w=1.2), however, the trends become complicated, depending on the multiplication factor of defectors r_d. When r_d becomes smaller, ε* sharply increases as the interior fixed point tends to disappear, the latter is shown by the dash line in Fig 2(d). Similar trends can also be observed under different combinations of α and β (Fig 2(f)).

3. I would also like to see figures S2 and S3 in the main text.

We thank the reviewer for this helpful suggestion. We have added sensitive analyses of N and θ under discretely changing environment w_1(t) in the main text. The manuscript has been modified as follows.

Page13(Fig 6)

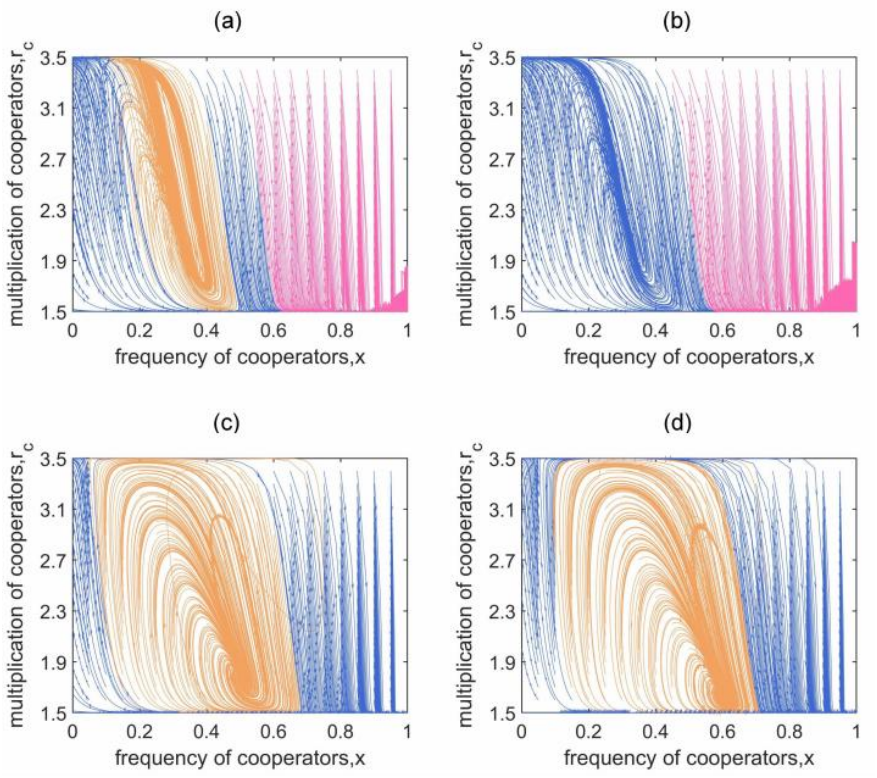

Fig 6. Game-environment evolution under different sizes of group N and distribution ratio of the expected total payoffs of the cooperators and defectors θ. Trajectories on x-r_c phase plane eventually evolve to x*=0, (x*=1, r_c*=α) or circulate along an interior closed orbit, which are distinguished by blue, pink and orange, respectively. Panels (a) and (b) correspond to N=5 and 6 with θ=0.5, r_d=0.6, T=2π and ε=9, while panels (a) and (c) correspond to θ=1 and 1.5 with N=4, r_d=1, T=10π and ε=6. In all panels, α=1.5, β=3.5.

Page12-13(Results)

In addition, we confirm whether the emergence of cyclic evolution is robust with regard to the changes of group size N and the distribution ratio of the expected total payoffs of cooperators and defectors θ. We find that the group size N could significantly affect the range of the orange region. Specifically, larger group size may hinder the emergence of cyclic evolution (Fig 6 (a)(b)), making it more difficult to reach the dynamic coexistence of cooperators and defectors. We also explore the influence of θ. Fig 6(c)(d) show that the final position of the interior closed orbit moves as θ changes, owing to the influence of θ on the fixed points of local game-environment evolution. The robustness of other parameters (such as the multiplication factor of defectors r_d and the range of cooperators' multiplication factor [α, β]), and the sensitivity analysis of the continuous model are also considered} (see Appendix S3).

Reviewer #3:

I thank the authors for their thorough response to the reviews and for meaningfully revising the manuscript to address these comments. The paper makes a meaningful contribution evolutionary game theory by integrating exogenous and endogenous environmental impacts on payoffs and thus dynamics.

We are very grateful for the encouraging and positive feedback and thanks for your constructive suggestions and your time.
